# Surface Enhanced Raman Spectroscopy for Quantitative Analysis: Results of a Large-Scale European Multi-Instrument Interlaboratory Study

**DOI:** 10.1021/acs.analchem.9b05658

**Published:** 2020-02-11

**Authors:** Stefano Fornasaro, Fatima Alsamad, Monica Baia, Luís A. E. Batista de Carvalho, Claudia Beleites, Hugh J. Byrne, Alessandro Chiadò, Mihaela Chis, Malama Chisanga, Amuthachelvi Daniel, Jakub Dybas, Gauthier Eppe, Guillaume Falgayrac, Karen Faulds, Hrvoje Gebavi, Fabrizio Giorgis, Royston Goodacre, Duncan Graham, Pietro La Manna, Stacey Laing, Lucio Litti, Fiona M. Lyng, Kamilla Malek, Cedric Malherbe, Maria P. M. Marques, Moreno Meneghetti, Elisa Mitri, Vlasta Mohaček-Grošev, Carlo Morasso, Howbeer Muhamadali, Pellegrino Musto, Chiara Novara, Marianna Pannico, Guillaume Penel, Olivier Piot, Tomas Rindzevicius, Elena A. Rusu, Michael S. Schmidt, Valter Sergo, Ganesh D. Sockalingum, Valérie Untereiner, Renzo Vanna, Ewelina Wiercigroch, Alois Bonifacio

**Affiliations:** † Raman Spectroscopy Lab, Department of Engineering and Architecture, University of Trieste, P.le Europa 1, 34100 Trieste, Italy; ‡ Université de Reims Champagne-Ardenne, BioSpecT-EA7506, UFR de Pharmacie, 51 rue Cognacq-Jay, 51097 Reims, France; § Faculty of Physics, Babes-Bolyai University, M. Kogalniceanu 1, 400084 Cluj-Napoca, Romania; ∥ Molecular-Physical Chemistry R&D Unit, Department of Chemistry, University of Coimbra, 3004-535 Coimbra, Portugal; ⊥ Chemometrix GmbH, Södeler Weg 19, 61200 Wölfersheim, Germany; # FOCAS Research Institute, Technological University Dublin, Kevin Street, Dublin 8, Ireland; ∇ Department of Applied Science and Technology, Politecnico di Torino, C.so Duca degli Abruzzi 24, 10129 Torino, Italy; ○ School of Chemistry, Manchester Institute of Biotechnology, University of Manchester, Manchester, United Kingdom M1 7DN; ◆ Radiation and Environmental Science Centre, FOCAS Research Institute, Technological University Dublin, Kevin Street, Dublin 8, Ireland; ¶ Jagiellonian Centre for Experimental Therapeutics, Jagiellonian University, ul. Gronostajowa 2, 30-384 Krakow, Poland; + Mass Spectrometry Laboratory (MSLab), MolSys RU, University of Liège, Liège, Belgium; ■ Univ. Lille, Univ. Littoral Côte d’Opale, EA 4490 – PMOI, F-59000 Lille, France; ▲ Centre of Excellence for Advanced Materials and Sensing Devices, Division of Materials Physics, Rudjer Boskovic Institute, Bijenicka c. 54, 10000 Zagreb, Croatia; ⬢ Department of Biochemistry, Institute of Integrative Biology, University of Liverpool, Liverpool, United Kingdom, L69 7ZB;; ≑ Bionanotechnology Research Section, Department of Pure and Applied Chemistry, University of Strathclyde, 99 George Street, Glasgow, G1 1RD, United Kingdom; ∞ Institute on Polymers, Composites and Biomaterials, National Research Council of Italy, via Campi Flegrei, 34, Pozzuoli, Naples 80078, Italy; ⊗ Nanostructures and Optics Laboratory, Department of Chemical Sciences, University of Padova, Via Marzolo 1 - 35131, Padova, Italy; ◨ Department of Life Sciences, University of Coimbra, 3000-456 Coimbra, Portugal; ★ Nanomedicine and Molecular Imaging Lab, Istituti Clinici Scientifici Maugeri IRCCS, Via Maugeri 4, 27100 Pavia, Italy; $ Technical University of Denmark, Department of Health Technology, Ørsteds Plads, Building 345C, DK-2800 Kgs. Lyngby, Denmark; % Silmeco ApS, Kenny Drews Vej 101, 2450 Copenhagen, Denmark; & Faculty of Health Sciences, University of Macau, SAR Macau, China

## Abstract

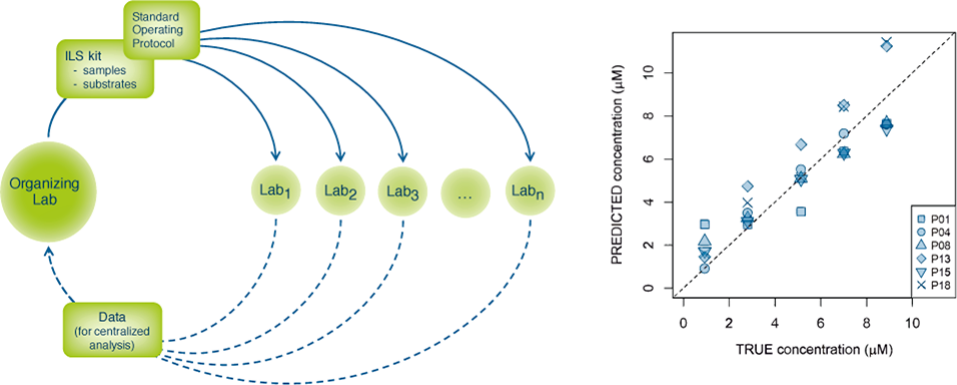

Surface-enhanced Raman scattering (SERS) is a powerful and sensitive technique for the detection of fingerprint signals of molecules and for the investigation of a series of surface chemical reactions. Many studies introduced quantitative applications of SERS in various fields, and several SERS methods have been implemented for each specific application, ranging in performance characteristics, analytes used, instruments, and analytical matrices. In general, very few methods have been validated according to international guidelines. As a consequence, the application of SERS in highly regulated environments is still considered risky, and the perception of a poorly reproducible and insufficiently robust analytical technique has persistently retarded its routine implementation. Collaborative trials are a type of interlaboratory study (ILS) frequently performed to ascertain the quality of a single analytical method. The idea of an ILS of quantification with SERS arose within the framework of Working Group 1 (WG1) of the EU COST Action BM1401 Raman4Clinics in an effort to overcome the problematic perception of quantitative SERS methods. Here, we report the first interlaboratory SERS study ever conducted, involving 15 laboratories and 44 researchers. In this study, we tried to define a methodology to assess the reproducibility and trueness of a quantitative SERS method and to compare different methods. In our opinion, this is a first important step toward a “standardization” process of SERS protocols, not proposed by a single laboratory but by a larger community.

Surface-enhanced Raman scattering (SERS) is a powerful and sensitive technique for the detection of fingerprint signals of molecules and for the investigation of a series of surface chemical reactions.^1^ Several monographs and reviews describe the mechanisms of SERS, confirming that metal (mostly silver and gold) nanostructures can generate a strong local electromagnetic field upon illumination with light having a wavelength capable of exciting localized surface plasmons. It is generally agreed that this electromagnetic mechanism (EM), as well as the chemical mechanism (CM), occurring when a chemical bond is formed between the metal and the adsorbed analyte, lead to the Raman signal enhancement of those analytes located close to or directly adsorbed onto the metal surface.^2^ In spite of its established sensitivity, SERS applied to quantitative analysis is still very challenging,^3−6^ and there is no general consensus on the key factors affecting performance.^7^ A number of studies have previously been designed to address some important issues regarding signal (enhancement) variability in SERS studies.^8−11^ Guicheteau et al.^10^ undertook an extensive, collaborative study within the U.S. to design and implement an evaluation protocol for SERS active surfaces enabling the definition of an enhancement value by which different substrates can be directly compared. However, there are other factors that cannot be easily disentangled from the substrate-related issues, such as the characteristics of the laser light used for excitation, the protocol for sample preparation (i.e., the way the analyte is put into contact with the metal surface), or the type of approaches used for data preprocessing and regression analysis. Muehlethaler et al.^11^ undertook a systematic study of such different aspects of the analytical procedure, in an attempt to qualitatively validate the SERS technique for forensic purposes, albeit in a single laboratory environment. Independently, many studies introduced quantitative applications of SERS in various fields, such as quantification of biomarkers, drugs and related metabolites in biofluids,^12−22^ or the determination of pesticides or toxins in foodstuffs or other biological samples.^23−25^ Several SERS substrates have been designed and tested for each specific application. Thus, among the published methods, there is a wide range in performance characteristics, analytes used, instruments, and analytical matrices.^6,26,27^ In general, very few methods have been validated according to international guidelines.^28^ In the literature, the evaluation of figures of merit (when performed) has been limited, as the validation protocols of the analytical method included only one piece of equipment/laboratory. The robustness of results is seldom assessed. As a consequence, the application of SERS in highly regulated environments is still considered risky, and the perception of a poorly reproducible and insufficiently robust analytical technique has persistently retarded its routine implementation outside academia. On the other hand, guidelines concerning the validation of analytical procedures, as detailed in official documents (regulatory or normative),^29^ do not explicitly cover the application of SERS, or even related techniques such as normal Raman spectroscopy. Collaborative trials (also called method performance studies) are a type of interlaboratory study (ILS) frequently performed to ascertain the performance (generally expressed as repeatability and reproducibility) of a single analytical method.^30^ As a part of the full method validation process, collaborative trials are very powerful tools to prove that an analytical method is indeed doing what it is supposed to do, independent of the laboratory in which the test is performed.^31^ Various examples can be found in the literature, especially for chromatographic techniques, but so far, to the best of our knowledge, such data have never been published for quantitative SERS, even if some examples can be found for qualitative methods.^11^ In this context, the use of arbitrarily defined criteria based on the experience of laboratories is a viable option.^32^ The idea of an ILS of quantification with SERS arose within the framework of Working Group 1 (WG1) of the EU COST Action BM1401 Raman4Clinics^33^ in an effort to overcome the problematic perception of quantitative SERS methods by addressing the following two questions: (i) Given the simplest conditions (i.e., a well-defined, well-known analyte in a simple matrix such as a buffered aqueous solution), can a quantitative SERS method be consistently implemented by different laboratories? (ii) If different SERS methods are used to quantify the same analyte, which is the best way to compare them?

These two general questions need to be further clarified. First, one must clearly define what a “SERS method” is. SERS signals of the same analyte strongly depend on the type of metal surface and on the choice of laser excitation wavelength.^34^ Therefore, the “complete” definition of a SERS method should take into account both a specific metal nanostructure (i.e., the substrate) and a specific laser wavelength (e.g., colloidal Ag excited at 785 nm, for brevity we use “cAg@785 nm”) as well as further working conditions. In this way, a method is completely described by the related “standard operating procedure” (SOP); ours are detailed within the accompanying Supporting Information. Second, the above question (i) actually consists of two distinct aspects: reproducibility and trueness (the meaning of both terms and their relation to accuracy, according to ISO 5725, are described in the Experimental Section). In other words, when a SERS method is applied by different laboratories using different instrumental setups, how similar to each other are the obtained results (reproducibility)? And how “close” to the truth are the obtained results (trueness)? We would like to stress that, at this stage, the reproducibility (also referred to as “precision”) is more important than the trueness, since reproducibility is usually considered the main concern for SERS methods. Once reproducibility has been assessed, trueness can be considered as well, as both are aspects of the overall accuracy for a method (see Table 1). In light of these clarifications, the second question (ii) can be better rephrased as, can one compare two or more SERS methods to assess which is the most reproducible?

**Table 1:** A Summary of the Figures of Merit Used for Method Characterization^a^

characteristic	description	FoM	interpretation
accuracy	closeness of agreement between measurement results and the accepted reference values	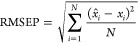	total prediction error
trueness	difference between the expected measurement results and the accepted reference values	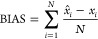	average of the residuals; systematic component of the total error (i.e., constant offset)
precision	closeness of agreement between independent measurement results obtained with the same method in different measurement facilities with different operators using different equipment	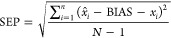	width of residuals distribution; random component of the total error

a *x̂*_*i*_ and *x*_*i*_ are the predicted and assigned reference values, respectively, for the test sample *i*, and *N* is the number of samples in the TEST set. Accuracy = reproducibility + trueness (RMSEP^2^ ≅ SEP^2^ + BIAS^2^), as from ISO 5725 (ref (39)).

To answer these questions, we set up an ILS in which six different SERS methods, involving Ag and Au plasmonic nanostructures (both colloidal and solid substrates), have been considered for the determination of adenine concentrations, chosen as the standard analyte (see Experimental Section for the justification of this choice). On one hand, we are well aware that the results of such a study are bound to be specific to the systems considered (i.e., adenine on very specific substrates), so that no extrapolation can yet be made to other substrates or analytes. We also expect the results to have a broader significance going beyond the system studied, especially as far as the methodology proposed to assess and compare SERS methods is concerned.

## Experimental Section

**Figure 1:**
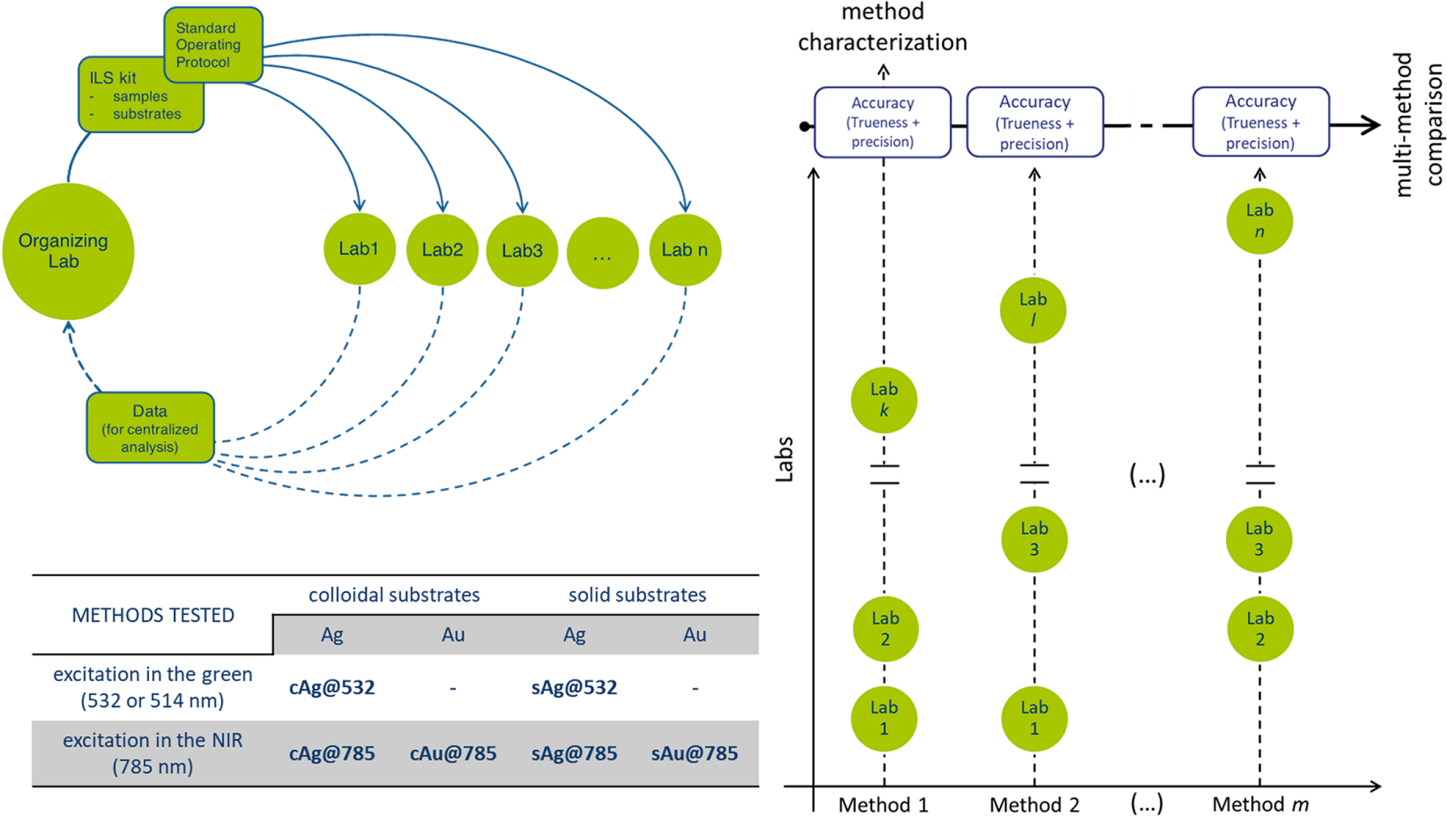
Organization structure of the Raman4Clinics WG1 interlaboratory study (ILS).

After the voluntary enrolment of the participating institutions from the membership of Raman4Clinics, the ILS started by collectively defining the study design, which was conclusively decided during a COST-Raman4Clinics WG1 meeting in Trieste in March 2018. The discussion eventually led to a study design in which six SERS methods were tested, and each method was independently evaluated by up to eight laboratories on the basis of the same SOP, using the same centrally provided materials for sample preparation. Samples and substrates were sent from the ILS organizing laboratory (OL, University of Trieste) to all participants, who sent all the data collected back to the OL for centralized data analysis (Figure 1). The results were combined to estimate the reproducibility obtained using the same protocol in different laboratories (along with trueness and accuracy) by calculating the corresponding Figures of Merit (FoMs, see Table 1 for a complete description). An SOP was prepared by the ILS OL and shared and agreed upon among the participants. The SOP detailed all the experimental procedures to be carried out by each ILS participant, specifying how to prepare the samples for analysis, how to perform a measurement, and how to export data. All laboratories were given a chance to provide feedback on the SOP, and revisions were introduced into a final document. For brevity, only the essential elements of the methodology are summarized here; for a more detailed description, see the Supporting Information (section S1, Standard Operating Procedure).

**Figure 2:**
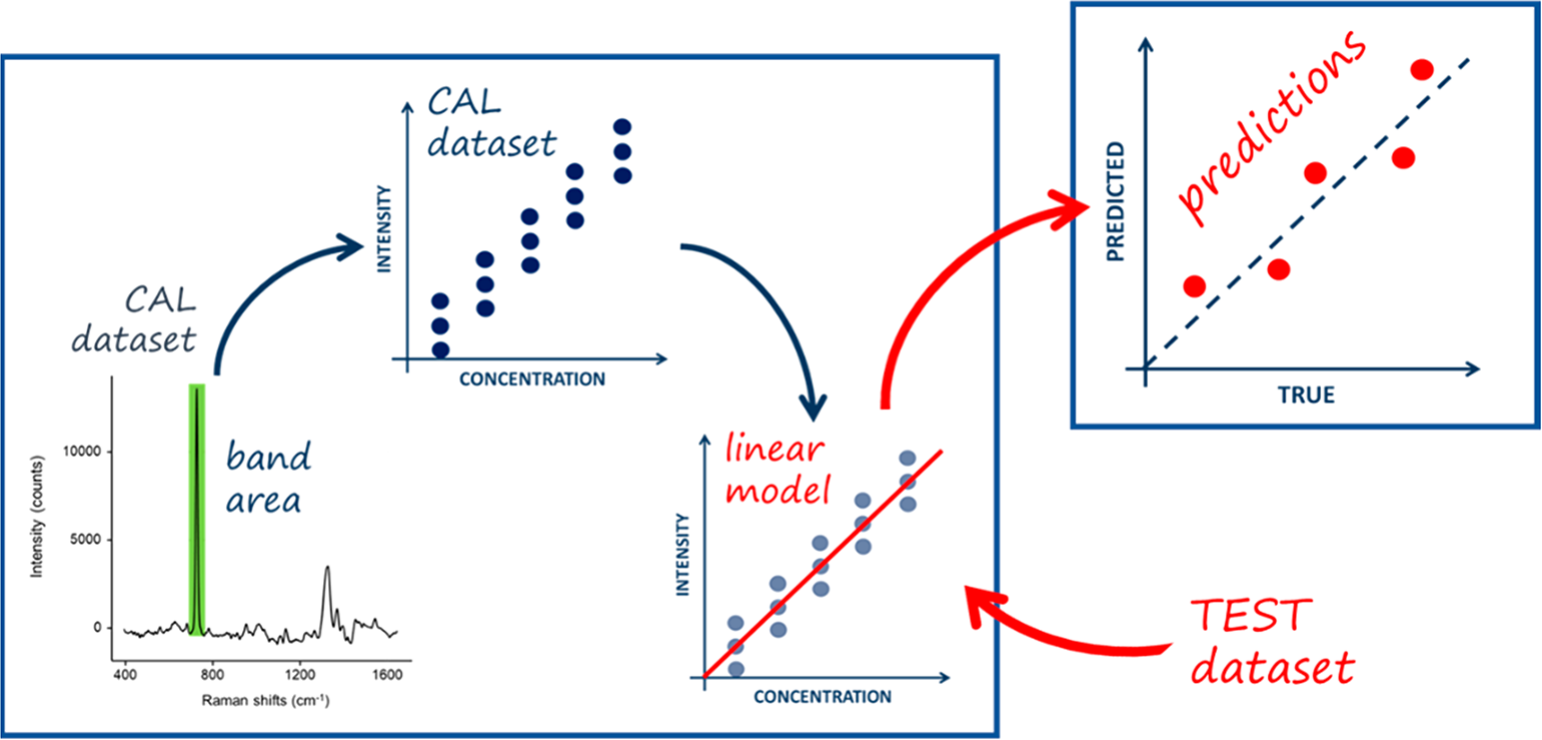
Single experiment validation scheme.

Each participant received a kit containing the necessary materials to prepare the samples and the SERS substrates necessary to perform the measurements, as detailed in the SOP. To ensure homogeneity, the kits were all assembled by the OL, using the same reagents and materials, and shipped to all ILS participants (Figure 1). For each experiment, all the participants prepared one calibration set (to build the regression model) and one test set (to validate the regression model and to compare the results) of samples (Figure 2). They performed SERS measurements using their own setups and instruments and sent the raw spectral data back to the OL. Only the OL knew the concentration of the test samples. The period for active participation was from July to October 2018 (see Supporting Information Section S3).

### Samples

All chemicals were acquired, aliquoted, and shipped by the OL. The selection of the analyte and of the SERS substrates to be used by the participants was mainly based on experience within the SERS community, the feasibility of performing experiments, and the availability of substrates. Selection criteria for the reference analyte included the ability to be detected by all the tested methods (and thus to adsorb on both Ag and Au substrates); the presence of intense, well-characterized bands in the SERS spectrum; the stability to light and temperature (to avoid complications during shipping and sample preparation); the absence of toxicity (to simplify international shipping and sample handling); the absence of tautomers under the measurement conditions (to ensure a SERS spectrum that was as simple as possible); the absence of thiol groups in the molecule (which lead to a very specific and strong metal–sulfur covalent bonds, usually not present in many analytes of interest such as drugs); the absence of electronic transitions in resonance with the excitation wavelengths used in the study (to allow the use of different excitation wavelengths while ruling out resonance Raman effects); and commercial availability at a reasonable cost. Eventually, after testing a short list of several substances, the choice fell on adenine as the standard analyte, while the choice of the matrix fell on pH 7.4 phosphate buffer (0.01 M), to ensure a constant pH and considering the availability of easily shipped buffer tablets ready to be dissolved. It should be stressed that many reasons behind the choice of adenine as the analyte are exclusively practical (e.g., stability, nontoxicity, affinity for both Ag and Au). It is very likely that other analytes would have been a better choice in terms of performance, but they lacked other characteristics which were deemed necessary given the available resources. The reader is referred to section S1, part 3, for a detailed description of the sample preparation.

### SERS Methods

The discussion on the selection of the SERS substrates to be used involved organizational aspects, such as the total number of samples, sustainability and reproducibility of metal nanostructures synthesis, and number of participants. There are several criteria to classify SERS substrates (e.g., top-down or bottom-up synthetic methods, surface characteristics, etc.), but most of them fall into one of two broad categories: colloidal or noncolloidal substrates (usually referred to as solid substrates). Everyone involved agreed that both these types of substrates should be included in the study. All participants were given the option of contributing with their own substrates, but eventually only one type of colloidal substrate and one type of solid substrate were offered as available for such a large number of experiments. As colloidal substrates, naked Ag and Au nanoparticles obtained by laser ablation synthesis in solution^35^ were provided by the University of Padova. Silmeco provided their commercially available Ag and Au solid substrates,^36^ based on metal-coated silicon nanopillars. The six SERS methods considered for this ILS are reported in Figure 1. The 785 and 532 nm excitation wavelengths were selected as the most commonly available among ILS participants. One participant had a 514 nm laser instead of a 532 nm, but on the basis of the previous experience within the working group, such a difference has been considered negligible, especially as nonresonant molecules were analyzed.

### SERS Measurements

Details on how to perform the measurements can be found in the SOP (section S1, parts 4.1 and 4.2), but we think it is particularly relevant to briefly report here some details about the number of measurements performed for each method. For colloidal substrates, three different batches of colloids for each metal were used, and participants were asked to collect, for each sample, three replicates using the three different batches (i.e., three spectra, one for each batch). For solid substrates, the official indication from the producer was to acquire a full map of several tens of spectra for each substrate. However, this would have translated into a substantial amount of work for each participant. Instead of a full map, the final version of the SOP prescribed the collection of three spectra (from random locations) for each substrate, and to use three different substrates for each sample, for a total of nine spectra for each sample (calibration or test).

### Instruments

**Figure 3:**
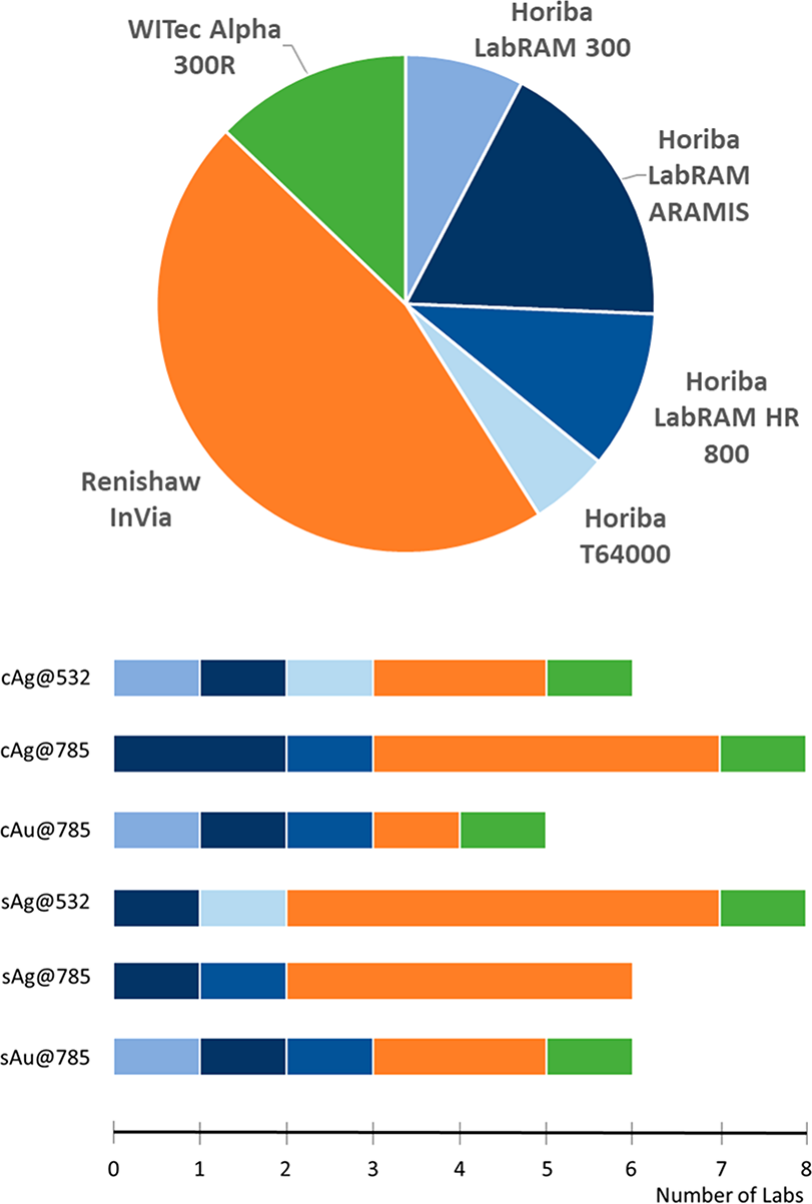
Instruments used in the study.

Six different models of Raman instruments were used in this ILS, from three different manufacturers (Figure 3). The instruments used were all calibrated according to the finalized protocol (section S1, part 4.3). Given the variety of setup characteristics used in the study, we decided to leave the choice of instrumental parameters such as laser power and acquisition time to each participant, as far as the signal-to-noise ratio of the sample containing the smallest amount of analyte was acceptable, suggesting upper thresholds of the laser power density and proper optical magnification (10× or 20×) for each method, to minimize potential sample photodegradation (see Supporting Information, section S1).

### Data Analysis

All statistical analysis and strategic decisions related to data analysis were entirely the responsibility of the OL; this was decided by all participants to be the most objective way of assessing reproducibility among the different laboratories. Calculations were performed within the R software environment (www.R-project.org, version 3.4.3) for statistical computing and graphics, building on the packages *hyperSpec*(37) and *dplyr*,^38^ on a commercially available workstation (Intel Core i7-4770, four-core 3.40 GHz, 32 GB DDR3-RAM). In-house developed R scripts (R code available upon request) were used for visualization and further processing.

Once all raw spectral data were collected, they were processed according to the following multistep procedure. Raw data were first inspected for data integrity (e.g., missing data, spectral artifacts) and then grouped and processed by a single experiment. The second step involved the preprocessing of the spectral data (e.g., smoothing, down-sampling, selection of spectral range, baseline correction, and normalization) and the building of a calibration curve by means of an inverse least-squares regression. The Supporting Information (section S2, Data Analysis Protocol) lists all relevant setting parameters necessary to reproduce the analysis in a software-independent manner and provides detailed information describing the protocol used in this step. Briefly, the inverse calibration approach was used under the assumption that the uncertainty coming from the preparations of spiked standards was negligible if compared with the random variability of each instrument. The calibration function was obtained using the calibration standards, relating the response (integrated area of SERS intensity between 715 and 750 cm^–1^) and the concentration (Figure 2).

Visual inspection of the calibration and residual plots, together with examination of the regression statistics obtained from each calibration curve (*r*^2^, F-test), was used as a system suitability check to make an overall assessment of the reliability of the data. If the fitness-for-purpose of the curve was judged to be satisfactory, each calibration model was further validated by plotting the predicted values versus the nominal concentrations of the test samples. A good calibration leads to observations falling close to a 45-degree straight line (*y* = *x* equality line). The results were visually examined by looking at the dispersion at each concentration level in the predicted versus reference values plot. These profiles are the visual decision tools that allowed us to evaluate the presence of possible different levels of precision for the considered methods and recognize regions with different levels of prediction accuracy between different laboratories.

To characterize and compare the performance of different SERS methods, during the next step of analysis, the residuals generated from multilaboratory predictions were grouped by method, and a set of performance measures, expressed as specific FoMs, was computed by taking into account the relationships between accuracy, trueness, and reproducibility (see Table 1). According to the ISO definition (ISO 5725),^39^ the overall accuracy of a method is considered as a global entity with two components, trueness and precision, representing the systematic and the random components of the total error, respectively.^40^ In the case of ILS, the precision is more appropriately expressed as reproducibility, defined as the difference between repeated measurements when between laboratories variations are included in each replicate. In this study, the root mean squared error of prediction (RMSEP) takes account of the simultaneous combination of the random and the systematic parts of the error that occur between the different laboratories, including errors from sample preparation, measurement, and the calibration model. The basic model is
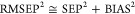
where the RMSEP is the estimate of the accuracy, the standard error of performance (SEP) accounts for the interlaboratory reproducibility (residual variance), and BIAS is the estimate of the trueness.^41^ It is worth noting that, when one compares the RMSEP, BIAS, and SEP obtained from different analyte ranges, rescaling can be advantageous, for example if the considered analytical protocols require different dilution steps. Here, we normalized RMSEP, BIAS, and SEP values using the range of the concentration values of the calibration references.

## Results and Discussion

The first notable achievement of the current study was the high number of participants throughout the European SERS community who accepted to share their expertise (creation and adoption of the consensual SOP) and experimental data (the first study to compare the reproducibility of different SERS methods). Fifteen laboratories from 11 European countries participated in the study, using six different models of Raman instruments from three manufacturers. Detailed technical information on each instrumental configuration that may lead to identification of the participating laboratories will not be disclosed. An aggregated summary is presented in Table 2.

**Table 2:** ILS in Numbers

laboratories involved	15
researchers involved	44
European countries involved	11
SERS protocols tested	6
single SERS substrates used	1080
metal colloids used	488 mL
spectra delivered	3694
spectra analyzed	3516

Of the 48 expected data sets, 41 (i.e., 85.4%) were uploaded by the participating laboratories, a total of 3694 spectra. The failure to upload data in the specified time window by three participants was mainly due to problems with the instrumentation or misjudgment of the time and effort required for carrying out the experiments. After data inspection, cAg@532 and cAu@785 data from laboratory P10 were rejected, because spectral features of parafilm, used as a hydrophobic substrate for liquid samples prepared from colloidal substrates (see SOP in Supporting Information, section S1), were found to strongly interfere with the adenine bands, impeding any data analysis. This problem may occur in cases of an incorrect focusing on the sample and could have been prevented by introducing a warning in the SOP, or by using more expensive substrates such as CaF_2_ slides, which were not available to all participants. After this first step, 39 data sets (3516 spectra), corresponding to 81.3% of the data sets planned, were further preprocessed (see Supporting Information Figure S2) and analyzed.

**Figure 4:**
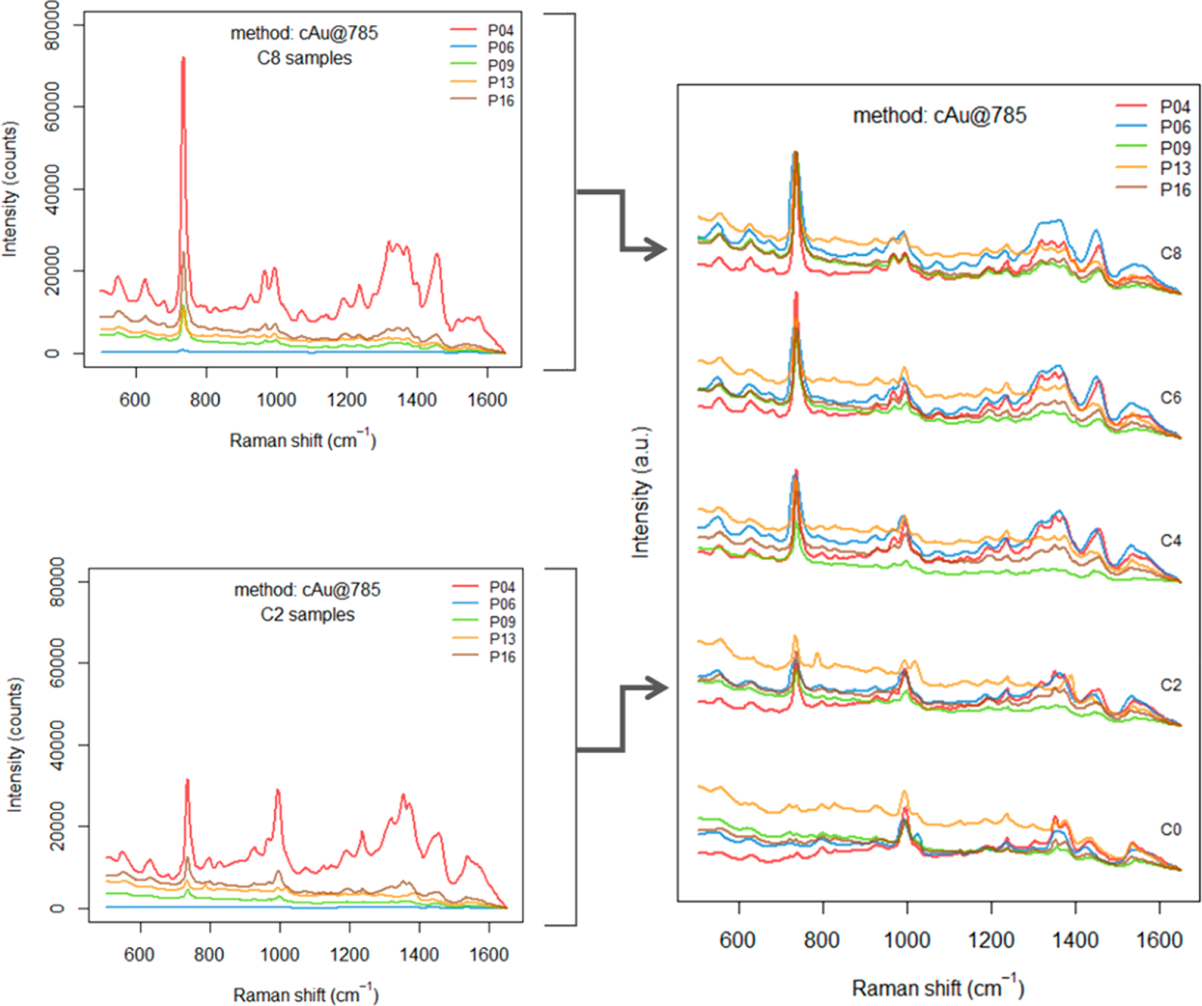
Comparison of spectra collected by different participants (P04, P06, P09, P13, and P16) at five concentration levels (C0, C2, C4, C6, C8) for the cAu@785 method (see Supporting Information, section S4, Table S1 for the actual concentrations and more details). On the left, spectra are shown before preprocessing. On the right, spectra are shown after preprocessing, offset for clarity.

After preprocessing, the specific ring-breathing mode of adenine observed in the range between 715 and 750 cm^–1^ appeared well resolved and nearly superimposable for spectra of samples of the same concentration. A specific example (method cAu@785) was selected and is shown in Figure 4 for illustration. The slight deviations among the spectra on the left side of Figure 4 can be attributed to variations in the laser power density, optics, and detectors’ responsivities, as produced by different instrumental setups used for the measurements. In fact, the peculiarities of each setup prevented the use of perfectly homogeneous measurement settings, and only a set of thresholds for maximum laser power densities have been suggested (see section S1 of Supporting Information). The preprocessing used for data analysis, which prescribed an extended multiplicative scatter correction (EMSC) of the spectra (see section S2 of Supporting Information, DAP), made possible the comparison between different data sets over the same concentration levels (Figure 4, on the right).

**Figure 5:**
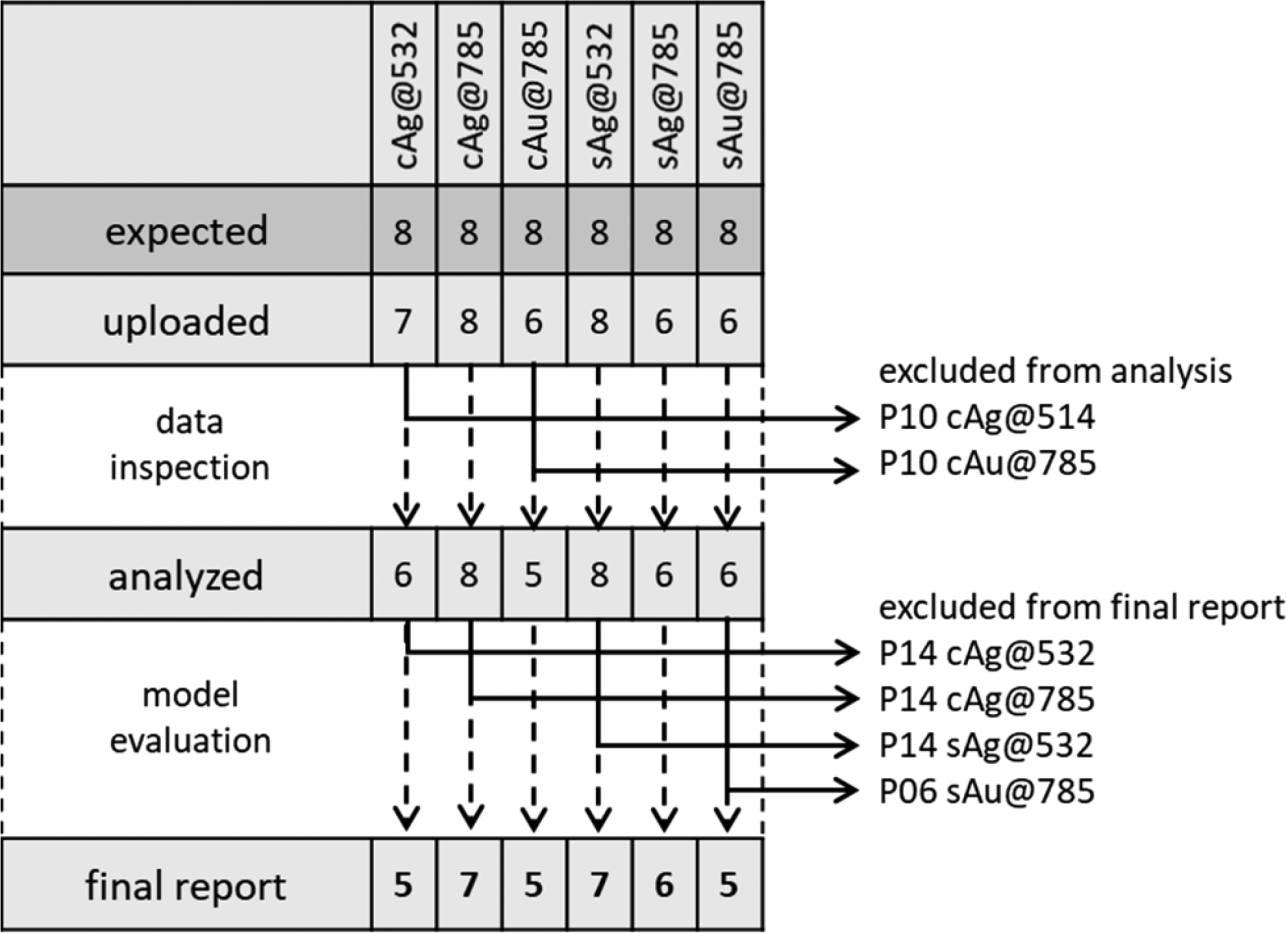
Data selection workflow.

The quantification of the SERS vibrational signature of adenine has been conducted by integrating the area for the region 715–750 cm^–1^ in the spectra from each training set and using it for the construction of the pertinent linear regression models by inverse least-squares regression. The performance and uncertainty of 39 calibration curves were analyzed for all laboratories. A simple linear model (straight line) fitted on the training data was selected because it is the most commonly accepted (and the most widely used) for other physicochemical analytical methods.^42^ The main requirement for this kind of model to be valid is that the computed values be sufficiently free of random errors to obtain a relationship able to give results that are proportional to the analyte concentration within a given range. Four calibration curves (cAg@532, cAg@785, and cAu@785 from laboratory P14 and sAu@785 from laboratory P06) were rejected because of very low quality of the linear fit (*r*^2^ lower than 0.6, *p* value for the *F* test higher than 0.01; see Supplementary Figure S3). The final data set was then composed of 35 curves, with a different number of laboratories for each method. Figure 5 shows the flowchart of the data evaluation strategy and the results obtained after the selection process. The entire data set can be found in the Supporting Information, section S4 Tables, sorted by methods and laboratory. Experimental data were deposited in the Zenodo data repository (https://zenodo.org/) and are publicly available (access number: 3572358).

Although ILS are usually designed to characterize just one method, we wanted to compare the reproducibility of more methods, each involving the use of the most commonly employed substrate types (i.e., colloidal and solid) and laser wavelengths (i.e., visible and near-infrared). Considering the number of participants and methods, the definition of an acceptable workload for each lab led to a design in which each method was going to be tested by a different group of laboratories. Collaborative trials are also very time-consuming (guidelines suggest at least eight valid results from different laboratories for each method). Although the study had been planned to achieve such numbers (i.e., eight laboratories for each method), we eventually managed to acquire and use only 35 data sets, of the 48 expected (i.e., 72.9%), leading to less than eight data sets for each method (Figure 5), and this may limit the strength of the obtained results. Preliminary trials with fewer laboratories, however, are still considered useful and are suggested by many international guidelines,^43^ especially for setting up a clearly written SOP that includes system suitability checks to be evaluated for errors and ambiguities before the actual collaborative trial starts. Thus, the results obtained in this study are to be considered as somewhat preliminary and will hopefully encourage other collaborative trials on SERS.

### Single Method Characterization

**Figure 6:**
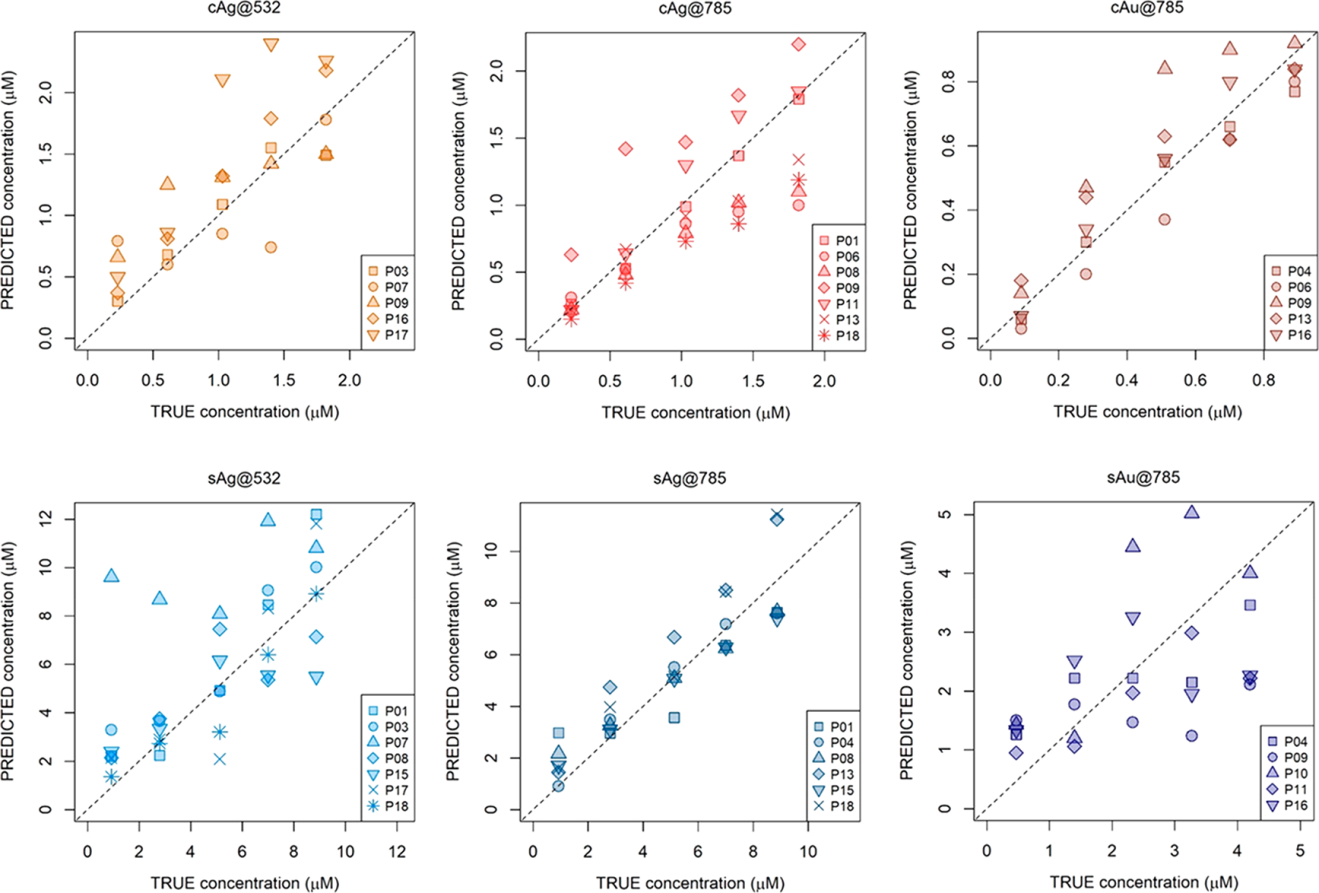
Plot of estimated values against the actual values for the test set samples for different methods by different participants (P01–P18). The dotted line is the line of equality. For a complete representation of the uncertainty of each individual prediction, see Figures S4–S9 in the Supporting Information.

The goal of calibration models is to predict the analyte concentration in an unknown (chemical) sample from instrument responses. The results from the predictions of the 35 selected calibration curves are summarized in Figure 6, presented as method-wise reference versus predicted value plots. Five adenine concentrations, over four different ranges, are covered. A visual inspection of this plot qualitatively describes the accuracy of each method. Although it appears that there is room for improvement, the predicted values were, in most cases, consistent. At least one laboratory for five of the considered methods obtained excellent results. The best performances were achieved for both colloids and solid substrates, within the cAg@785 (P01) and sAg@785 (P04) methods.

The spread in the adenine predictions at each concentration level represents the variability among the laboratories. In three cases (cAg@532, cAu@785, sAg@785), the predictions show a certain level of homoscedasticity, or in other words, the prediction error seemed independent of the concentration. In the other three cases (cAg@785, sAg@532, sAu@785), the spread increased as the concentration increased. More insights can be obtained from Figure S10 of the Supporting Information: in the three methods with a common internal variance, the median prediction error at each concentration level lies on the diagonal. Interestingly, many perfectly good curves in the cAg@785 method exhibit symptoms of slight nonlinearity (P06, P08, P13, P18), with the errors at the right end tending to curve away from the equality line. It should be noted that the instrumental differences were not influential at all, since they were performed with different instruments (cf. Figure 3).

**Figure 7:**
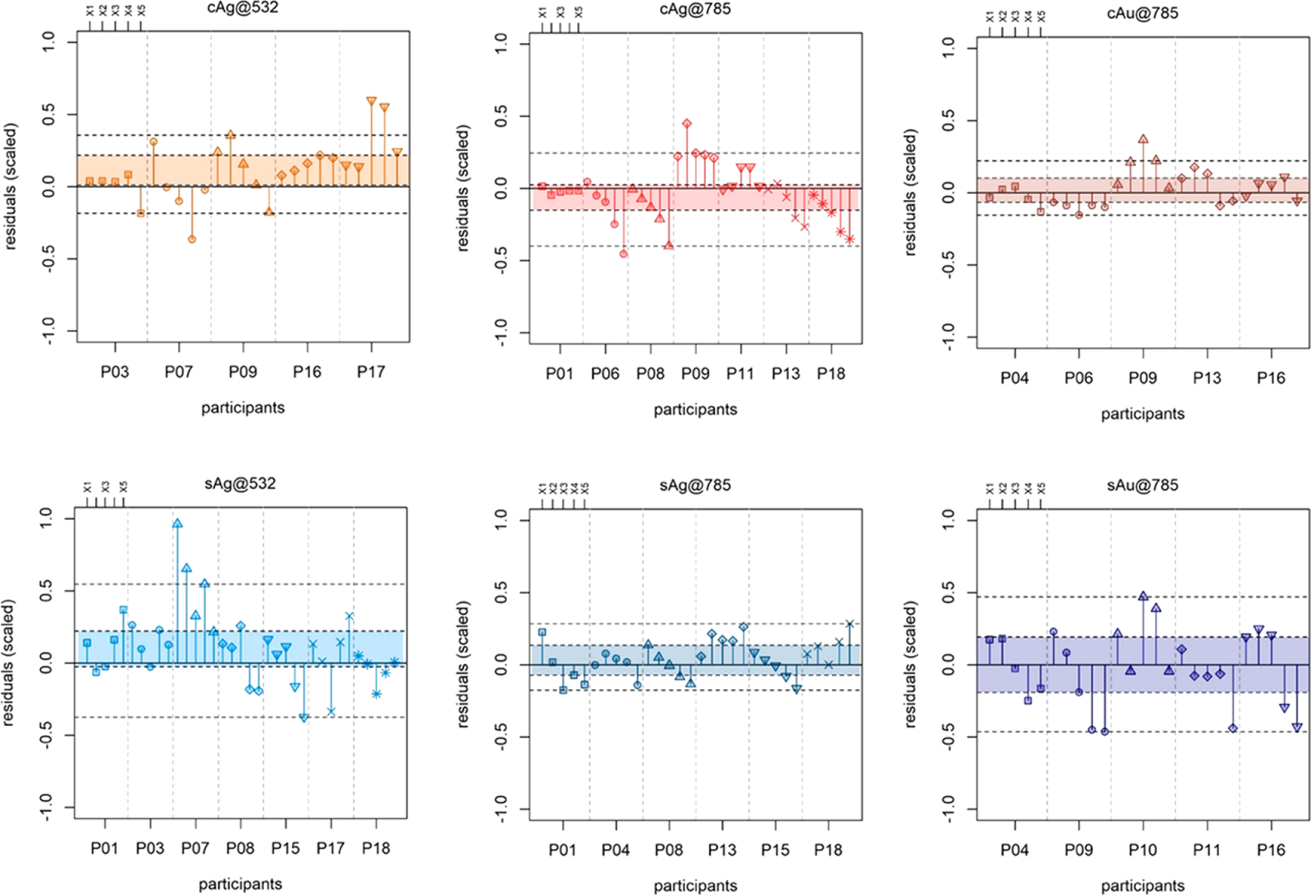
Residual plots for the TEST sets of the six SERS methods. Concentration levels of the TEST sets (X1–X5; for actual concentrations, see Supporting Information, Table S1) are aggregated according to each participant (P01–P18). The limits of the colored areas are the upper and lower quartiles, so each area spans the interquartile range (IQR) for each method; The two dashed lines outside the colored areas range to the extreme data point (<1.5 × IQR). Residuals values were rescaled using the range of the reference values in the test set data to enable comparison between calibrations obtained with different ranges.

The residual plots in Figure 7 summarize the results from another perspective by focusing on the normalized prediction errors (i.e., residuals): each group represents a different laboratory, whereas the points within each group represent the five concentration levels for the test sets (X1–X5). In addition, colored areas are shown, corresponding to the interquartile range (IQR) and two control lines (<1.5 × IQR), calculated over the entire set of residuals for each method. These plots are a visual decision tool that allows the evaluation of the discrepancies between different laboratories without the need for tests of significance. The larger the IQR, the larger the data deviation is, indicating poorer the performance. Moreover, severe outliers are immediately identified. However, the outliers were not excluded from the FoMs calculation because such rejection would have only artificially improved the appearance of the data but do nothing in terms of avoiding future instances of outlying results.

### Comparison among Methods

To gain more insight into the consistency of quantitative results obtained from different SERS methods, we computed a set of performance measures, expressed as specific FoMs calculated from the residuals from the validation samples. A summary is provided in Table 3.

**Table 3:** Figures of Merit for Different SERS Methods^a^

method	*N*	RMSEP	SEP	BIAS
cAg@532	25	24%	21%	11%
cAg@785	35	19%	19%	–4%
cAu@785	25	13%	13%	3%
sAg@532	35	29%	27%	11%
sAg@785	30	13%	12%	4%
sAu@785	25	28%	29%	–2%

a Normalization (as range scaling) was carried out to compare different methods; non-normalized RMSEP, BIAS, and SEP values are available in Supporting Information Table S8 (see Methods for details). *N* is the number of residuals for each method.

**Figure 8:**
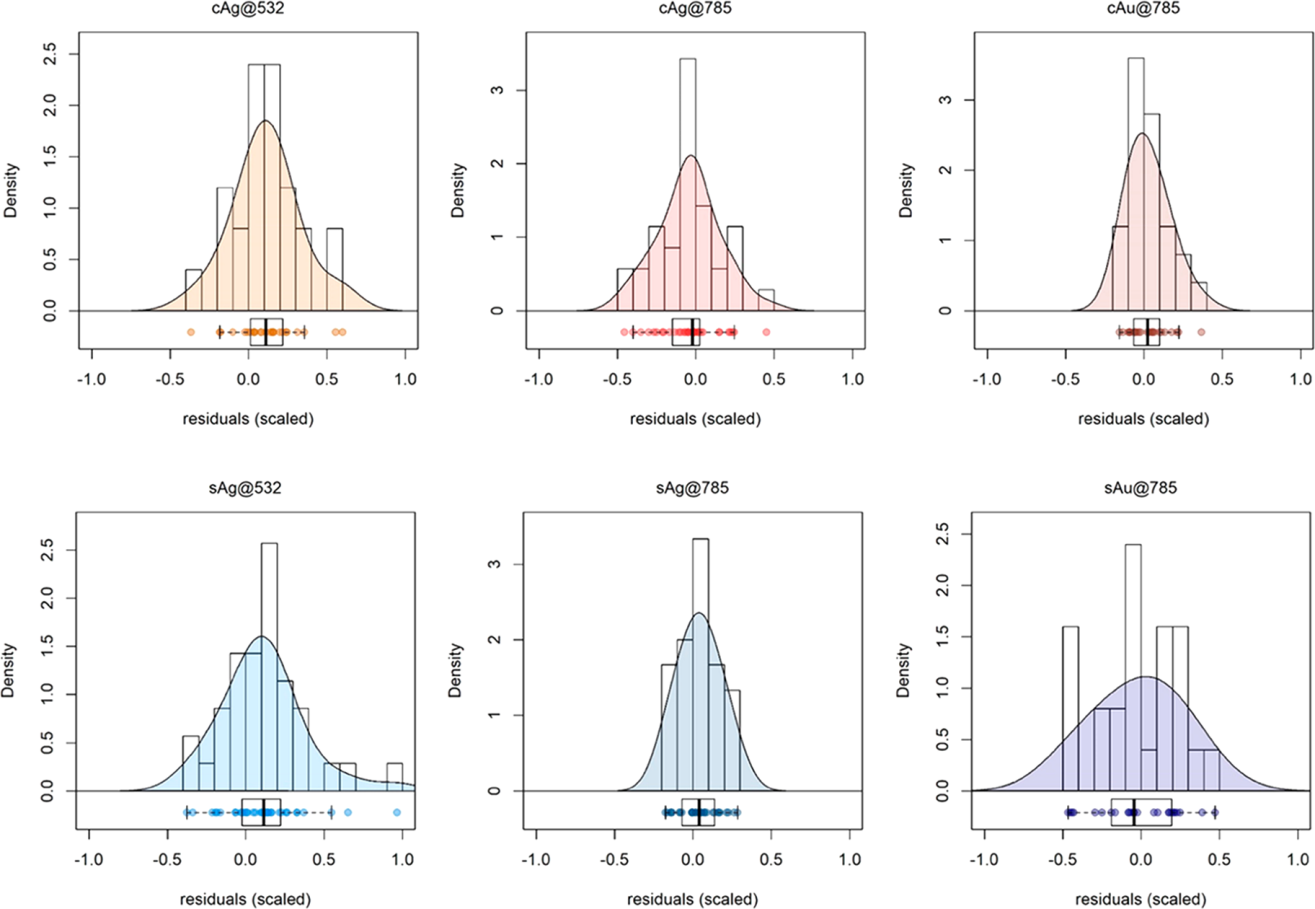
Probability density function (PDF) and boxplots of the normalized residuals for the six SERS methods.

The general behavior of the residuals for each SERS method is depicted in Figure 8. For all the considered methods, the residuals have a distribution that looks roughly normal in shape and centered close to zero. A failure to center on zero is described as a bias, and the size of the mean error is the BIAS value, calculated as the average difference between predicted and reference samples in the validation set. The width of the distribution is described by the SEP value, calculated as the square root of the quadratic sum of the error of the predicted versus the reference value, once the predicted value has been corrected for bias (Table 1).

A high BIAS means a low trueness of the methods. The main concern here is with the possible importance of the calculated BIAS, since it is the nature of spectroscopic data to present some variation occurring between measurements due to randomly distributed noise. A Student’s *t* test at the 99% confidence level verified whether these deviations could be considered random noise, as usual and expected, or whether the deviations were larger than expected by random chance alone. Only two methods (both employing the 532 nm laser source, cAg@532 and sAg@532) demonstrated significant BIAS in the selected range.

In terms of reproducibility, two methods, i.e., sAg@785 and cAu@785, appeared as the most reliable, with narrower distribution of residuals and SEP values of 12% and 13%, respectively.

Interestingly, the inverse of the (normalized) SEP is often used as a quality threshold for model performance.^44,45^ For 1/SEP > 4, the calibration is considered acceptable for sample screening; for 1/SEP > 10, the calibration is considered acceptable for quality control; and for 1/SEP > 15, the calibration is good for quantification. In this context, all methods could be considered acceptable for screening purposes, with the exception of sAg@532 and sAu@785 (1/SEP = 3.72 and 3.49, respectively). Considering the fact that this is the first collaborative trial on quantitative SERS conducted on a range of different instrumental setups, the fact that two methods, one using a colloidal substrate (i.e., cAu@785) and one using a solid substrate (i.e., sAg@785), achieved a 1/SEP value close to the limit set for quality control (i.e., 1/SEP > 10) is significant.

Although the primary focus of this study is on reproducibility, the RMSEP, used as a practical measure of accuracy, enabled us to characterize a SERS method by a single acceptability criterion, consistent with the ISO definition. The lower the RMSEP, the better the method is. The RMSEP typically express how well a calibration, on average, will predict new samples. This study, however, was conducted to compare the performance of a whole analytical process, from sample preparation to the stability of the instrument, for which the calibration is just a part. As expected from the low values of BIAS, the overall accuracy as indicated by the RMSEP is reflecting the SEP values, indicating two methods (i.e., cAu@785 and sAg@785) as the most accurate.

It must be noted that the RMSEP of a method, as calculated in this study, includes all the uncertainty contributions from different laboratories, depending essentially on the design of the experiments presented in the SOP. Since the RMSEP is calculated from all test samples, thus averaging over different laboratories, it does not directly provide an uncertainty for future measurements by a single lab, but it has been used here exclusively to compare the accuracy of different methods.

### Limitations and Possible Improvements

As with all first steps, this study is still imperfect, amendable, and somewhat limited in scope. The overall results in themselves are to be strictly considered as limited to prescribed methods using specific substrates to quantify adenine and should not be extended to SERS in general or to other methods or analytes. The aim was not even to quantify adenine (which was chosen out of necessity, having desirable characteristics of stability, nontoxicity, etc.; see Experimental Section) but to assess how different results were obtained among different laboratories and to be able to compare the performance of different methods. In a way, this study is more about methodology than performance. Moreover, it had the merit of having fostered active collaboration among tens of spectroscopists all over Europe, in an effort to reach a consensus on how to evaluate SERS experiments performed by different laboratories. Since SERS is increasingly being used by individual laboratories for quantitative applications, this issue is clearly extremely relevant to the SERS community, as proved by a very recent review written by a panel of international researchers that addressed this issue by proposing some recommendations, in terms of “good analytical practice,” to increase the comparability of quantitative SERS results obtained by different laboratories.^6^ In spite of these positive findings, even the SERS methods tested with the lowest SEP do not yet satisfy the strict reproducibility requirements for a quantitative analytical method (1/SEP > 15). However, there is space for improvement: the use of internal standards, whether these are isotopologues or through use of standard addition (as reviewed in ref (7)), could help to decrease the intrinsic variability due to the enhancement substrates. Although not within the goals of this study, it may be of interest to devote future effort to understanding the mechanistic rationale underlying the differing responses of the different substrates. For solid substrates, a larger data set including maps instead of single measurements could also improve the data. The use of nonlinear models could take into account deviations from linearity which appeared in many calibration data sets, thus improving the predictions.

## Conclusions

In this first SERS study involving several laboratories, we tried to define a methodology to assess the reproducibility and trueness of a quantitative SERS method and to compare different methods. In our opinion, this is a first important step toward a “standardization” process of SERS protocols, not proposed by a single laboratory but by a larger community. This study addressed two questions: can a quantitative SERS method consistently be used by different laboratories? And if different SERS methods are used to quantify the same analyte, which is the best way to compare them? On the basis of the results obtained, we suggest that indeed a SERS method can be consistently used by different laboratories, provided that the method is very well-defined (with a detailed SOP that all participants agree to follow). The methods tested provided varying results in terms of reproducibility, but the best ones proved to be reasonably reproducible, with an average SEP as low as 12% and 13%, which is promising considering the fact that different instruments were used over a wide time frame, with different setup and acquisition parameters (laser power, acquisition time, etc.). These results are valid within the framework of the system we proposed to use to compare different methods, considering RMSEP, SEP, and BIAS values in Table 3. Using these tools, one can effectively compare different SERS methods to assess which one is the more reproducible and accurate. The present study is a starting point and should ideally stimulate other groups of SERS researchers to set up similar studies for other analytes, substrates, and methods. The next step with respect to the present kind of study should be the evaluation of each source of experimental uncertainty (e.g., substrates, instruments, and operators) for the best performing methods, as already suggested for qualitative SERS methods.^11^ Future SERS studies should possibly focus their effort on a single method, rather than many, to reach more easily a significant number of laboratories.

## Supporting Information Available

Experimental Section: S1. Standard Operating Procedure; S2. Data Analysis Protocol; S3. Supplementary Figures; S4. Supplementary Tables (PDF)

## Accession Codes

The data set is available at DOI: 10.5281/zenodo.3572358, access number: 3572359.
