# Supplementary material for: Surface Enhanced Raman Spectroscopy for Quantitative Analysis: Results of a Large-Scale European Multi-Instrument Interlaboratory Study
Source: Anal Chem. 2020 Feb 11;92(5):4053–64. doi: 10.1021/acs.analchem.9b05658 (PMC7997108; doi:10.1021/acs.analchem.9b05658)
Supplement: Supplementary file 1 — ac9b05658_si_001.pdf [file ac9b05658_si_001.pdf]

Supplementary material for:

Surface Enhanced Raman Spectroscopy for quantitative analysis: results of a large-scale European multi-instrument interlaboratory study.

Stefano Fornasaro[a], Fatima Alsamad[b], Monica Baia[c], Luís A.E. Batista de Carvalho[d], Claudia Beleitese, Hugh J. Byrne[f], Alessandro Chiadò[g], Mihaela Chis[c], Malama Chisanga[h], Amuthachelvi Daniel[i], Jakub Dybas[j], Gauthier Eppe[k], Guillaume Falgayrac[l], Karen Faulds[o], Hrvoje Gebavi[m], Fabrizio Giorgis[g], Royston Goodacre[n], Duncan Graham[o], Pietro La Manna[p], Stacey Laing[o], Lucio Litti[q], Fiona M. Lyng[i], Kamilla Malek[j], Cedric Malherbe[k], Maria P.M. Marques[d,r], Moreno Meneghetti[q], Elisa Mitri[a], Vlasta Mohaček-Grošev[m], Carlo Morasso[s], Howbeer Muhamadali[n], Pellegrino Musto[p], Chiara Novara[g], Marianna Pannico[p], Guillaume Penel[l], Olivier Piot[b], Tomas Rindzevicius[t], Elena A. Rusu[c], Michael S. Schmidt[u], Valter Sergo[a,v], Ganesh D Sockalingum[b], Valérie Untereiner[b], Renzo Vanna[s], Ewelina Wiercigroch[j], Alois Bonifacio[a*].

[a] Raman Spectroscopy Lab, Department of Engineering and Architecture, University of Trieste, P.le Europa 1, 34100 Trieste, Italy; [b] Université de Reims Champagne-Ardenne, BioSpecT-EA7506, UFR de Pharmacie, 51 rue Cognacq-Jay, 51097 Reims, France; [c] Faculty of Physics, Babes-Bolyai University, M. Kogalniceanu 1, 400084 Cluj-Napoca, Romania; [d] Molecular-Physical Chemistry R&D Unit, Department of Chemistry, University of Coimbra, 3004-535 Coimbra, Portugal; [e] Chemometrix GmbH, Södeler Weg 19, 61200 Wölfersheim, Germany; [f] FOCAS Research Institute, Technological University Dublin, Kevin Street, Dublin 8, Ireland; [g] Department of Applied Science and Technology, Politecnico di Torino, C.so Duca degli Abruzzi 24, 10129 Torino, Italy; [h] School of Chemistry, Manchester Institute of Biotechnology, University of Manchester, Manchester, UK, M1 7DN; [i] Radiation and Environmental Science Centre, FOCAS Research Institute, Technological University Dublin, Kevin Street, Dublin 8, Ireland; [j] Faculty of Chemistry, Jagiellonian University, ul. Gronostajowa 2, 30-387 Krakow, Poland; [k] Mass Spectrometry Laboratory (MSLab), MolSys RU, University of Liège, Liège, Belgium; [l] Univ. Lille, Univ. Littoral Côte d'Opale, EA 4490 - PMOI, F-59000 Lille, France; [m] Centre of Excellence for Advanced Materials and Sensing Devices, Division of Materials Physics, Rudjer Boskovic Institute, Bijenicka c. 54, 10000 Zagreb, Croatia; [n] Department of Biochemistry, Institute of Integrative Biology, University of Liverpool, Liverpool, UK, L69 7ZB; [o] Bionanotechnology Research Section, Department of Pure and Applied Chemistry, University of Strathclyde, 99 George Street, Glasgow, G1 1RD, United Kingdom; [p] Institute on Polymers, Composites and Biomaterials, National Research Council of Italy, via Campi Flegrei, 34, Pozzuoli, NA 80078, Italy; [q] Nanostructures & Optics Laboratory, Department of Chemical Sciences, University of Padova, Via Marzolo 1 - 35131, Padova, Italy;[r] Department of Life Sciences, University of Coimbra, 3000-456 Coimbra, Portugal; [s] Nanomedicine and Molecular Imaging Lab, Istituti Clinici Scientifici Maugeri IRCCS, Via Maugeri 4, 27100 Pavia, Italy; [t] Technical University of Denmark, Dep. of Health Technology, Ørsteds Plads, Building 345C, DK-2800 Kgs. Lyngby, Denmark; [u] Silmeco ApS, Kenny Drews Vej 101, 2450 Copenhagen, Denmark; [v] Faculty of Health Sciences, University of Macau, SAR Macau, China.

Raman4Clinics – Work Group 1

Interlaboratory study

Contents

- S1 “Standard Operating Procedure”
    1. Overview
    2. WG1-RR Kit content
    3. Samples preparation
    4. Data acquisition
    5. Exporting/uploading data
- S2 “Data Analysis Protocol”
    1. Data Import/Loading
    2. Data Pre-processing
    3. Calibration
    4. Model Validation
- S3 “Supplementary Figures”
    1. Data upload
    2. Preprocessing
    3. Calibration evaluation
- S4 “Tables”
- References

Raman4Clinics – Work Group 1

Interlaboratory study

S1 - "Standard Operating Procedure"

1. Overview

IMPORTANT. Please read carefully ALL the procedures before starting the experimental activities.

Each Participant will receive a WG1-RR **kit** (see Section 2) containing

- paracetamol powder, required for setup characterization
- the necessary materials to prepare the analyte samples (CALIBRATION samples, and TEST samples)
- the SERS substrates (solid substrates or metal colloids) necessary to perform the measurements

Six possible SERS **METHODS** have been selected for this Round Robin test, each method being defined by a substrate and an excitation wavelength: **sAg@514/532**, **cAg@514/532**, **sAg@785**, **cAg@785, sAu@785**, **cAu@785**.

Each Participant will prepare the samples (see Section 3) and perform the measurements (see Section 4) according to the protocols defined in this Standard Operating Procedure (SOP), for each method assigned to her/his Lab (2 or 3 methods for each Participant).

Samples will be prepared by subsequent dilutions with PBS from stock solutions (see Section 3).

Measurements will be collected according to the following experimental design (see Section 4 for further details). For each method, the following samples will be made available:

1. a **CALIBRATION set** (10 samples C0, C1, …C9), to build the regression model and
2. a **TEST set** (5 samples X1, X2, … X5) to validate the model

Wherever possible, specific experimental parameters (*e.g. maximum laser power density, optics for illumination and collection*), will be provided (see Section 4).

Once the data have been collected, they will be submitted to a **centralized data analysis** (by the RR coordinator) to get a list of figures of merit (FOMs) characterizing the analytical performance for each calibration. Once the analysis is done, details on how the analysis has been performed will be made available to all Participants.

2. Wg1-RR Kit content

Each Participant will receive a **kit** (Figure 1) containing:

- 1 Eppendorf tube (labelled as **ADE**) with adenine (CAS-Nr.73-24-5) to be used for calibration[1].
- 1 Eppendorf tube (labelled as **X**) with adenine (CAS Nr. 73-24-5) to be used for validation[1].

IMPORTANT: upon receiving the kit, these tubes should be stored in the refrigerator at 4°C until used

- 1 small plastic Petri dish (labelled as **PBS**) with a phosphate buffer saline (PBS) tablet.
- 1 Eppendorf tube (labelled as **REF**) containing paracetamol (acetaminophen; CAS Nr. 103-90-2) powder.

And, depending on the type of measurements to be performed by the Participant:

- 3 Falcon tubes (labelled as **cAu #1, #2 and #3**) with 7 mL of Au colloid each *AND/OR*
- 3 Falcon tubes (labelled as **cAg #1, #2 and #3**) with 7 mL of Ag colloid each
- 1 sheet with the UV-vis extinction spectra of the colloids (*to be used as reference in* 4.1.1)

IMPORTANT: colloidal substrates should be stored in the dark at 4°C until used and SHOULD NOT BE FROZEN; with time, heavier particles could deposit on the bottom, vortex or shake the tube before use. Possibly, colloidal substrates should be used within 1-2 weeks from their arrival with the kit.

- 1 plastic envelope (labelled as **sAu**) with 45 Au solid SERS substrates *AND/OR*
- 1 plastic envelope (labelled as **sAg**) with 45 Ag solid SERS substrates

IMPORTANT: solid substrates should be stored at room temperature and in the dark until used; they are stored under vacuum: once opened, use within 1-2 weeks.

[1] The exact amount of adenine in the "ADE" and "X" tubes is only known by the RR Coordinator, and will be made known (together with the concentrations of all the solution samples) once the data are uploaded (see Data policies).

Interlaboratory study

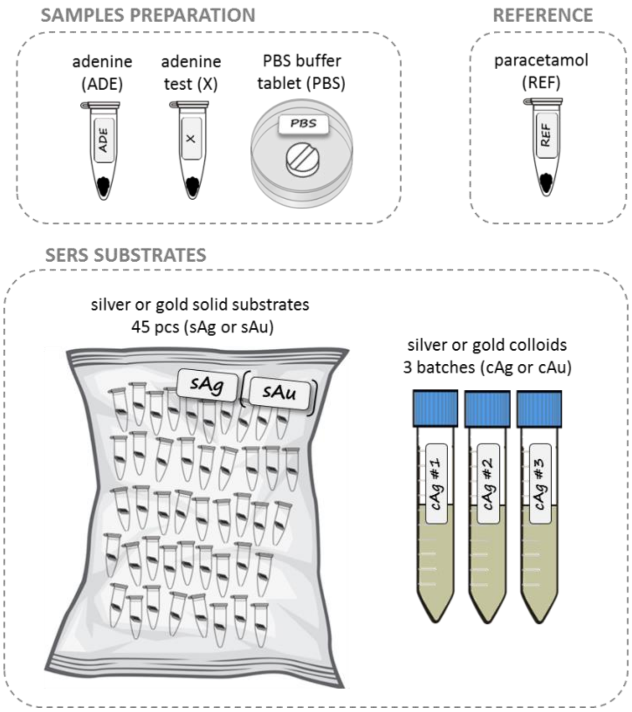

Figure 1 A schematic representation of the WG1-RR kit shipped to each Participant

3. Samples preparation

3.1. Materials/reagents needed

Before starting, make sure you have the following materials and reagents **(not included in the kit)**:

- common plastic and glass tubes/vessels, especially 1.5 -2.0 mL Eppendorf tubes
- volumetric flask (10 mL) – *(IMPORTANT: the use of a proper volumetric flask is needed to reduce experimental errors for dilutions)*
- laboratory balance
- NaOH (sodium hydroxide, CAS Nr. 1310-73-2) pellets
- milliQ/deionized water
- micropipettes - (*IMPORTANT: check their calibration before use, uncalibrated micropipettes can lead to significant experimental errors*)

To minimize experimental errors, volume measurements should be preferably performed using adequate micropipettes, which have recently been purchased or calibrated:

Table 1

volume to be measured	micropipettes to be used
10-20 μL	2-20 μL (**P20**)
20-100 μL	10-100 μL (**P100**)
100-200 μL	20-200 μL (**P200**)
200-1000 μL	100-1000 μL (**P1000**) pipette
1000-5000 μL	500-5000 μL (**P5000**) pipette

3.2. CALIBRATION samples preparation

3.2.1. Prepare the NaOH-analyte stock A solution by directly adding **740 μL** (using a P1000) of an aqueous solution of **NaOH 1M** (i.e. 1 mol NaOH in 1 litre of milliQ/deionized water) to the analyte in the **ADE Eppendorf tube** (Figure 2);

3.2.2. Prepare the **PBS buffer solution** by dissolving the **PBS tablet** in **200 mL of milliQ/deionized water**;

3.2.3. Prepare 10 mL of the PBS-analyte stock B solution: add 10 μL of the stock A solution (3.2.1) (using a P10 or a P20) to a 10 mL volumetric flask containing 5 mL of the **PBS buffer solution** (3.2.2); mix and then add **PBS buffer solution** until the final volume of 10 mL (Figure 2);

3.2.4. Prepare 10 mL of the PBS-analyte stock C solution: add 1 mL of the stock B solution (3.2.3) (using a P1000) to a 10mL volumetric flask containing 5mL of the **PBS buffer solution** (3.2.2); mix and then add **PBS buffer solution** until the final volume of 10 mL (Figure 2);

3.2.5. Prepare the analyte **CALIBRATION C0, C1, C2, … C9 samples** by diluting the stock B solution (3.2.3) or the stock C solution (3.2.4) with the **PBS buffer solution** (3.2.2) in Eppendorf tubes according to the dilution schemes (Tables 2a-c, page 7). Since different methods require samples covering different concentration ranges, each Participant will prepare a different C0-C9 series for each method assigned by the Coordinator, according to the proper dilution schemes in Tables 2a-c.

(EXAMPLE: P01 has been assigned 2 methods, cAg@785 and sAu@785; thus P01 needs to prepare and measure 2 series of CALIBRATION C1...C9 samples)

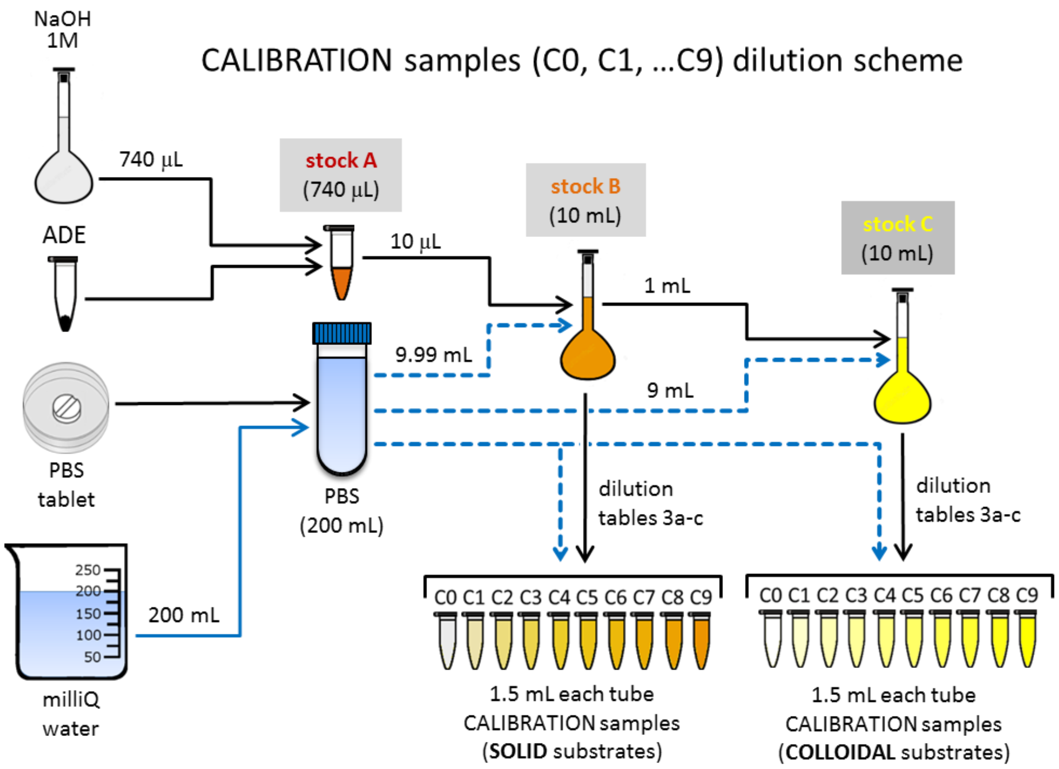

Figure 2. Scheme for the preparation of CALIBRATION samples.

*Table 2a. Dilution schemes for **CALIBRATION** samples **(Ag@541/532nm)***

**CALIBRATION** samples<br>Dilution schemes for methods using Ag substrates @514/532 nm				
analyte solution	**COLLOIDAL** cAg@514/532 nm		**SOLID** sAg@514/532 nm	
	stock C solution (μL)	**PBS-buffer solution** (μL)	stock B solution (μL)	**PBS-buffer solution** (μL)
**C0**	0	1500	0	1500
**C1**	60	1440	30	1470
**C2**	120	1380	60	1440
**C3**	180	1320	90	1410
**C4**	240	1260	120	1380
**C5**	300	1200	150	1350
**C6**	360	1140	180	1320
**C7**	420	1080	210	1290
**C8**	480	1020	240	1260
**C9**	540	960	270	1230

*Table 2b. Dilution schemes for **CALIBRATION** samples (**Ag@785nm**)*

**CALIBRATION** samples<br>Dilution schemes for methods using Ag substrates @785 nm				
analyte solution	**COLLOIDAL** cAg@785 nm		**SOLID** sAg@785 nm	
	stock C solution (μL)	**PBS-buffer solution** (μL)	stock B solution (μL)	**PBS-buffer solution** (μL)
**C0**	0	1500	0	1500
**C1**	60	1440	30	1470
**C2**	120	1380	60	1440
**C3**	180	1320	90	1410
**C4**	240	1260	120	1380
**C5**	300	1200	150	1350
**C6**	360	1140	180	1320
**C7**	420	1080	210	1290
**C8**	480	1020	240	1260
**C9**	540	960	270	1230

*Table 2c. Dilution schemes for **CALIBRATION** samples (**Au@785nm**)*

**CALIBRATION** samples<br>Dilution schemes for methods using **Au substrates** @**785** nm				
analyte solution	**COLLOIDAL** cAu@785 nm		**SOLID** sAu@785 nm	
	stock C solution (µL)	**PBS-buffer solution** (µL)	stock B solution (µL)	**PBS-buffer solution** (µL)
**C0**	0	1500	0	1500
**C1**	30	1470	15	1485
**C2**	60	1440	30	1470
**C3**	90	1410	45	1455
**C4**	120	1380	60	1440
**C5**	150	1350	75	1425
**C6**	180	1320	90	1410
**C7**	210	1290	105	1395
**C8**	240	1260	120	1380
**C9**	270	1230	135	1365

3.3. TEST samples preparation

3.3.1. Prepare the NaOH-analyte stock XA solution (unknown adenine concentration) by directly adding **740 µL** of an aqueous solution of **NaOH 1M** (i.e. 1 mol NaOH in 1 litre of milliQ/deionized water), using a P1000, to the analyte in the **X Eppendorf tube** (Figure 4);

3.3.2. Prepare 10 mL of the PBS-analyte stock XB solution: add 10 µL of the stock XA solution (3.3.1) (using a P10 or a P20) to a 10 mL volumetric flask containing 5mL of the **PBS buffer solution** (3.2.2); mix and then add **PBS buffer solution** until the final volume of 10 mL (Figure 4);

3.3.3. Prepare 10 mL of the PBS-analyte stock XC solution: add 1 mL of the stock XB solution (3.3.2) (using a P1000) to a 10 mL volumetric flask containing 5 mL of the **PBS buffer solution** (3.2.2); mix and then add **PBS buffer solution** until the final volume of 10 mL (Figure 3);

3.3.4. Prepare the analyte **TEST X1, X2, … X5 samples** by diluting the stock XB solution (3.3.2) or the stock XC solution (3.3.3) with the **PBS buffer solution** (3.2.2) in Eppendorf tubes according to the dilution schemes (Tables 3a-c, page 9). Since different methods require samples covering different concentration ranges, each Participant will prepare a different X1-X5 series for each method assigned by the Coordinator, according to the proper dilution schemes in Tables 3a-c.

(EXAMPLE: P01 has been assigned 2 methods, cAg@785 and sAu@785; thus P01 needs to prepare and measure 2 series of TEST X1...X5 samples)

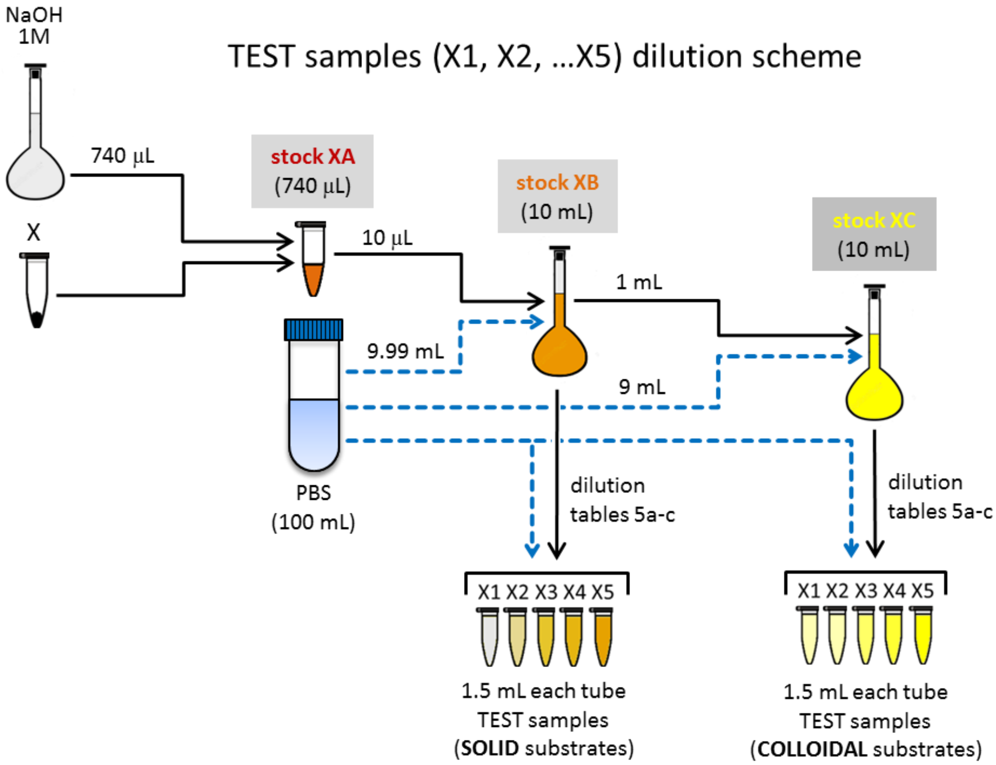

Figure 3 Scheme for the preparation of TEST samples.

Table 3a. Dilution schemes for ***TEST*** *samples (**Ag@514/532**)*

**TEST** samples<br>Dilution schemes for methods using **Ag substrates** @**514/532** nm				
**analyte solution**	**COLLOIDAL** cAg@514/532 nm		**SOLID** sAg@514/532 nm	
	stock XC solution (μL)	**PBS-buffer solution** (μL)	stock XB solution (μL)	**PBS-buffer solution** (μL)
**X1**	50	1450	20	1480
**X2**	130	1370	60	1440
**X3**	220	1280	110	1390
**X4**	300	1200	150	1350
**X5**	390	1110	190	1310

Table 3b. Dilution schemes for ***TEST*** *samples (**Ag@785**)*

TEST samples<br>Dilution schemes for methods using Ag substrates @785 nm				
analyte solution	**COLLOIDAL** cAg@785 nm		**SOLID** sAg@785 nm	
	stock XC solution (μL)	**PBS-buffer solution** (μL)	stock XB solution (μL)	**PBS-buffer solution** (μL)
**X1**	50	1450	20	1480
**X2**	130	1370	60	1440
**X3**	220	1280	110	1390
**X4**	300	1200	150	1350
**X5**	390	1110	190	1310

*Table 3c. Dilution schemes for **TEST** samples (**Au@785**)*

TEST samples<br>Dilution schemes for methods using Au substrates @785 nm				
analyte solution	**COLLOIDAL** cAu@785 nm		**SOLID** sAu@785 nm	
	stock XC solution (μL)	**PBS-buffer solution** (μL)	stock XB solution (μL)	**PBS-buffer solution** (μL)
**X1**	20	1480	10	1490
**X2**	60	1440	30	1470
**X3**	110	1390	50	1450
**X4**	150	1350	70	1430
**X5**	190	1310	90	1410

4. Data acquisition

4.1. SERS measurements with COLLOIDAL substrates

SUMMARY. For each sample (CALIBRATION or TEST), 1 spectrum must be acquired **with each** of the 3 different COLLOIDAL substrate batches (#1, #2 and #3) provided, for a total of 3 spectra/sample (see Figure 4). This means each Participant is expected to collect, **for each method using a COLLOIDAL substrate**, a total of 3 x 10 = 30 spectra for the CALIBRATION set, plus a total of 3 x 5 = 15 spectra for the TEST set, for a grand total of 45 spectra.

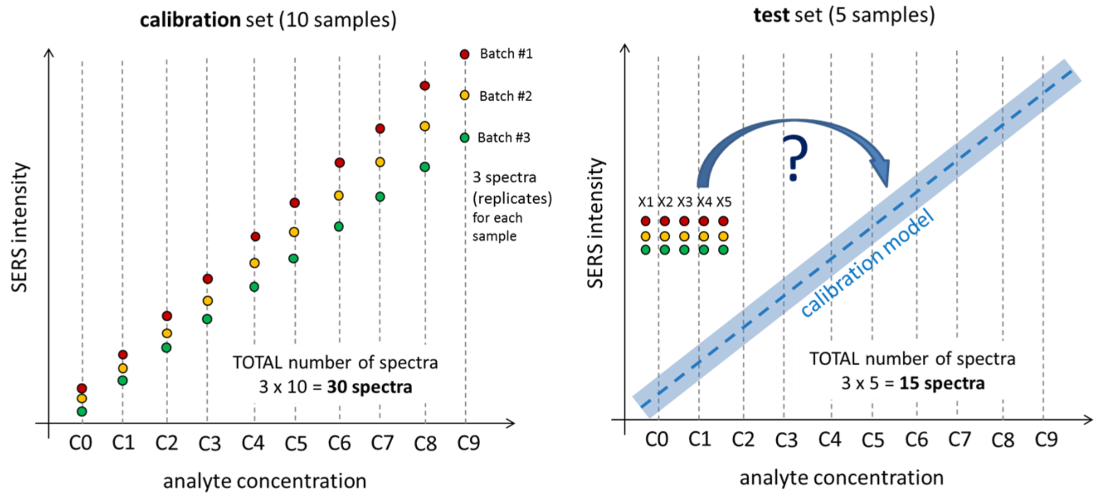

Figure 4 Overview of experimental design for COLLOIDAL substrates

4.1.1. **CHECK COLLOIDAL SUBSTRATES with UV-vis**. Before starting data acquisition, it is strongly advised that the Participant checks the colloidal substrates by collecting a UV-vis extinction spectrum and comparing it with the one of the freshly-prepared colloids included in the kit. To collect a UV-vis extinction spectrum using a quartz cuvette with a 10 mm path length, dilute the colloid with milliQ water by a ratio 1:4 (i.e. 1 part colloid, 4 part water). If 2 mm path length cuvettes are used, use undiluted colloids. *If the extinction spectrum collected presents significant differences in band maximum position or overall shape with respect to the spectrum of fresh colloids, contact the Coordinator.*

4.1.2. **PREPARE A SLIDE**. Take a glass microscope slide, cover it first with an aluminium foil (to avoid fluorescence from the glass) and then with Parafilm (to keep the surface hydrophobic and ensure the formation of a drop with a certain height, Figure 5.1 and 5.2). (ALTERNATIVE) Instead of these glass-aluminum-parafilm slides, UV-quality CaF_2 microscope slides can also be used.

4.1.3. **ADD SAMPLES TO SUBSTRATES**. Using a P100, add **25 μL** of a sample to **25 μL** of a COLLOIDAL substrate in a 1.5 mL Eppendorf tube and rapidly mix (few seconds); then immediately transfer the whole volume of the mixture (i.e. a 50 μL drop, P100) onto a slide under the microscope objective for data collection.

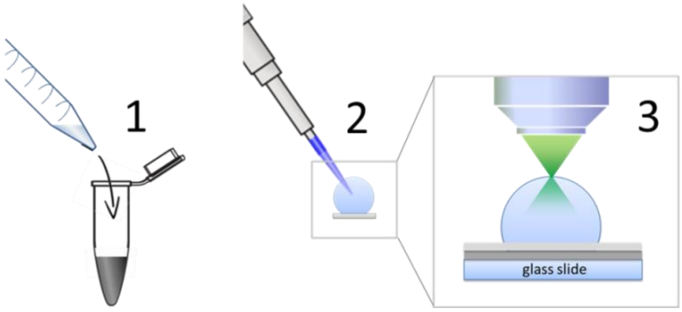

Figure 5 Sample preparation and data collection using a COLLOIDAL substrate

4.1.4. **ACQUIRE DATA.** To collect a spectrum, focus the laser beam onto the top of the deposited drop using a **10x** or **20x** objectives (Figure 5.3), ensuring that the laser power density at the sample does not exceed the values indicated below (Table 4). Collect the spectrum in the region 400-2000 cm^{-1}, adjusting collection parameters to maximize the signal-to-noise ratio. **The first sample to be analysed should be the C9**, which is expected to yield the most intense signal, taking care to adjust the laser power and the exposure time to avoid the saturation of the detector, while maximizing the intensity of the most intense band of adenine. The number of accumulated scans should be enough to ensure an excellent signal-to-noise ratio. Examples of spectra from C1 and C9 samples obtained with different methods are shown on page 16 (Figure 9). *Spectra should be saved with filenames according to the schemes detailed in Section 5.*

IMPORTANT: after collecting the spectrum for the C9 sample, data collection should be randomized, i.e. samples should NOT be measured in order of increasing or decreasing concentration.

Table 4. Upper thresholds of laser power density for COLLOIDAL substrates

COLLOIDAL substrates		
**laser wavelength**	**maximum power density* (W/cm²)**	
	**cAu**	**cAg**
514/532 nm	N.A.	$6 \cdot 10^4$ W/cm²
785 nm	$5 \cdot 10^3$ W/cm²	$5 \cdot 10^3$ W/cm²

* to calculate the laser power densities, the laser spot diameter (*d*) were derived from instrument specifications or estimated using the approximated formula ***d*** = (1.22·λ)/N.A.; the values reported were derived from real measurements.

4.2. SERS measurements on SOLID substrates

SUMMARY. For each sample (CALIBRATION or TEST), 3 spectra from different spots will be acquired **from each of 3 different, independent substrates** (in case of solid substrates), for a total of 9 spectra/sample (see Figure 6). This means each Participant is expected to collect, **for each method using SOLID substrate**, a total of 9 x 10 = 90 spectra for the CALIBRATION set, plus a total of 9 x 5 = 45 spectra for the TEST set, for a grand total of 135 spectra.

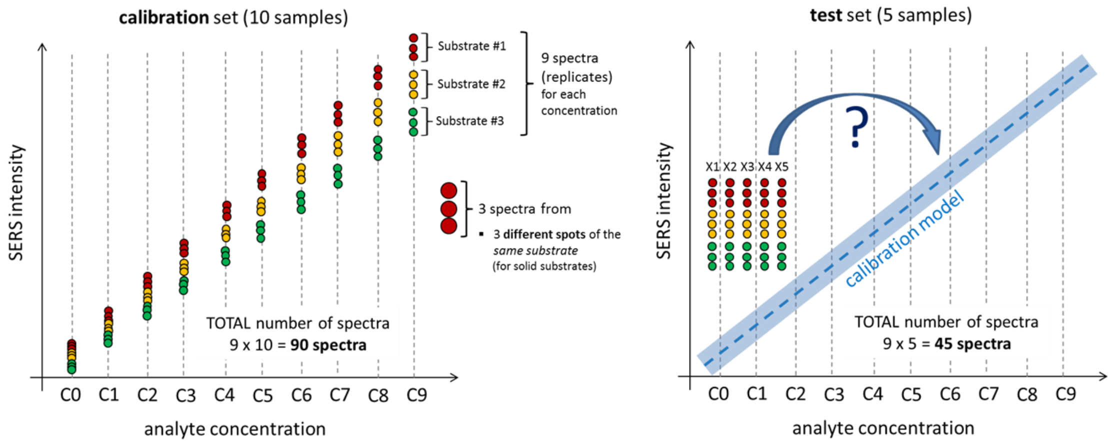

Figure 6 Overview of experimental design for SOLID substrates

4.2.1. **ADD SAMPLES TO SUBSTRATES.** Place the SOLID substrates on a glass microscope slide (dull-grey side face down, iridescent yellow-orange side face up), and deposit 15 μL of

sample onto each SOLID substrate (aiming at the centre of the substrate) using a P100, taking care not to touch the substrate surface with the pipette tip (Figure 7.1).

4.2.2. **RINSE SUBSTRATES**. Wait for the deposited sample drops to dry at room temperature (approximately up to a max of 1.5 h[2], see Figure 8) and, holding the substrates with a pair of tweezers, rinse them by rapidly dipping them into milliQ water (Figure 7.2) for 3 times (take extra care when handling the substrate with the tweezers)[3]. Let the remaining water on the substrate dry (*ca*. 30-45 min) before proceeding.

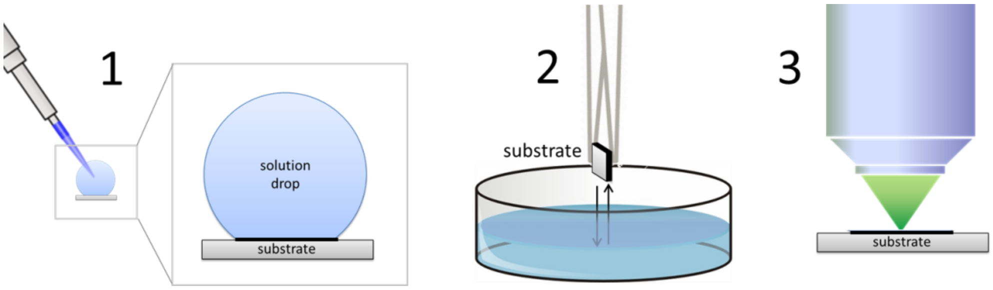

Figure 7 Sample preparation and data collection using a SOLID substrate

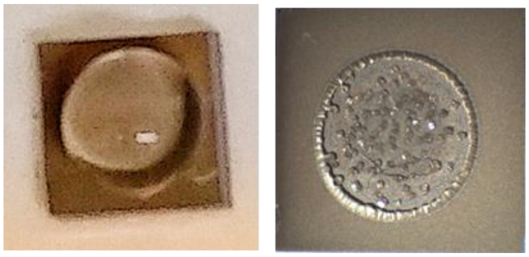

Figure 8. Sample drop on a substrate, before (left) and after (right) drying.

4.2.3. **ACQUIRE DATA.** To collect a spectrum, focus the laser beam onto the substrate using a **10x** or **20x** objectives (Figure 7.3), ensuring that the laser power density at the sample does not exceed the values indicated below (Table 5). Collect the spectrum in the region 400-2000 cm^{-1}, adjusting collection parameters to maximize the signal-to-noise ratio. **The first sample to be analysed should be the C9**, which is expected to yield the most intense signal, taking care to adjust the laser power and the exposure time to avoid the saturation of the detector,

[2] Some Participant reported longer times.
[3] Upon drying, buffer crystals are forming on the substrates. Even after the triple washing, crystals tend to leave some kind of "footprints" (i.e. you can still tell where the crystals were). These do not interfere with the measurements.

while maximizing the intensity of the most intense band of adenine. The number of accumulated scans should be enough to ensure an excellent signal-to-noise ratio. **For each substrate, 3 spectra should be collected from different spots.** Examples of spectra from C1 and C9 samples obtained with different methods are shown on page 16 (Figure 9)[4]. *Spectra should be saved with filenames according to the schemes detailed in Section 5.*

OPTIONAL: once the 3 spectra from random spots on a substrate are collected according to the protocol above, Participants are welcome to acquire small maps as additional data.

IMPORTANT: after collecting the spectrum for the C9 sample, data collection should be randomized, i.e. samples should NOT be measured in order of increasing or decreasing concentration.

Table 5. Upper thresholds of laser power density for SOLID substrates.

SOLID substrates		
**laser wavelength**	**maximum power density* (W/cm^2)**	
	**sAu**	**sAg**
514/532 nm	N.A.	$2 \cdot 10^5$ W/cm^2
785 nm	5×10^3 W/cm^2	$2 \cdot 10^4$ W/cm^2

* to calculate the laser power densities, the laser spot diameter (*d*) were derived from instrument specifications or estimated using the approximated formula ***d*** = (1.22·λ)/N.A.; the values reported were derived from real measurements.

[4]. The spectra at page 16 are all from Ag substrates, SERS spectra of adenine on Au substrates are slightly different than those on Ag, so don't worry if you "get more bands" than the spectra on the SOP.

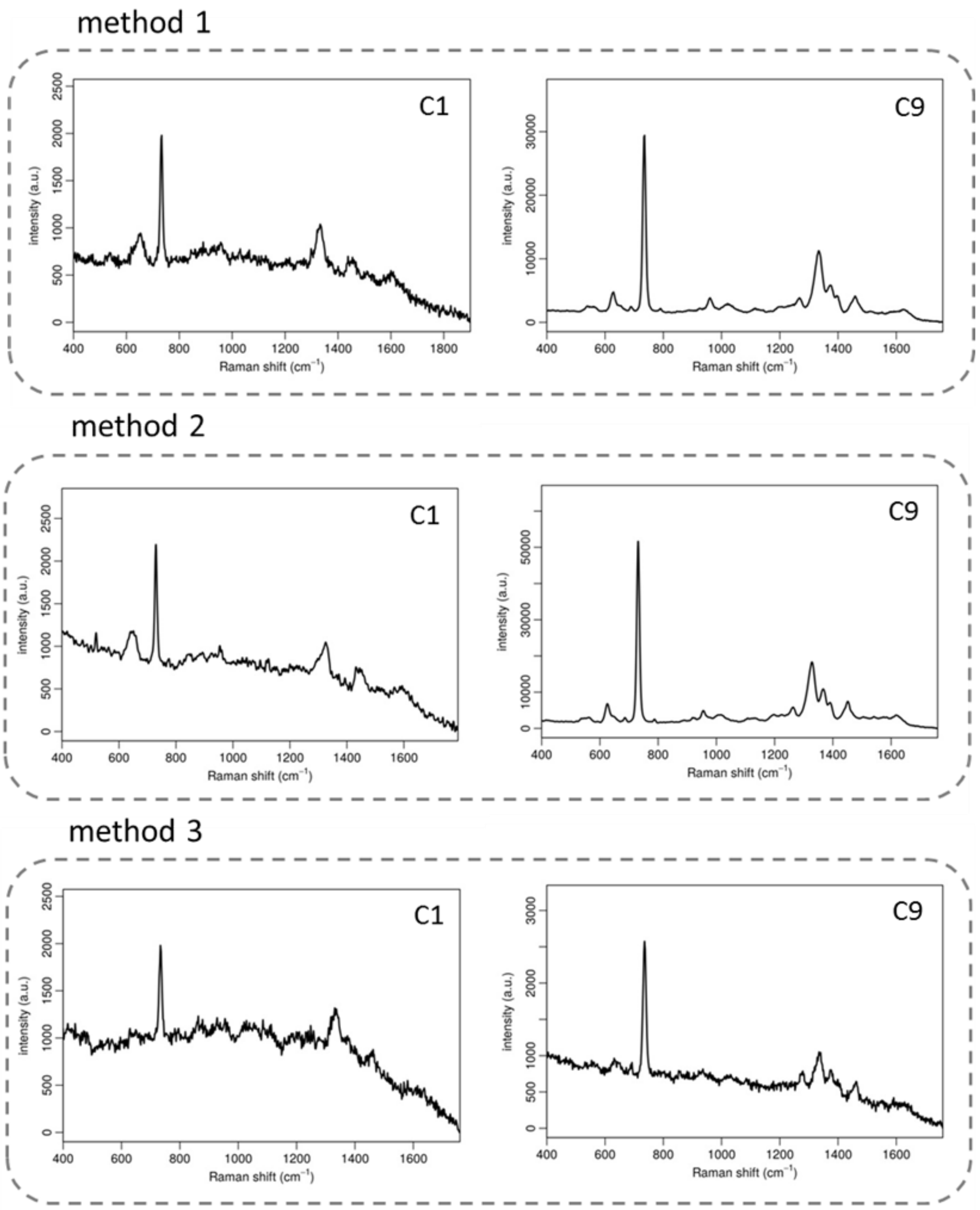

Figure 9 Examples of unprocessed adenine spectra from C1 and C9 samples (i.e. from lowest/highest concentrations) obtained with different methods (i.e. with different substrate/laser combinations).

4.3. Characterization of the experimental setup

After the collection of the spectra from samples, acquire, **for each method used for sample analysis**, a normal Raman spectrum of the **REF** powder in the region 400-2000 cm^{-1}, using the **same optics and laser power used for the SERS measurements** (as in 4.1-4.2), but **increasing the exposure time or the number of accumulated scans by a factor of 10**. The spectrum will be used to characterize each setup in terms of wavenumber calibration and sensitivity.

(EXAMPLE: to collect spectra of the samples for the method sAg@785nm, Participant P01 used a 20x objective, a laser power at the sample of 20 mW, and exposure time of 10s and accumulated 2 scans; to collect the spectrum of the REF powder, P01 will use the same conditions but with 20 scans accumulations OR increasing the exposure time to 100s.)

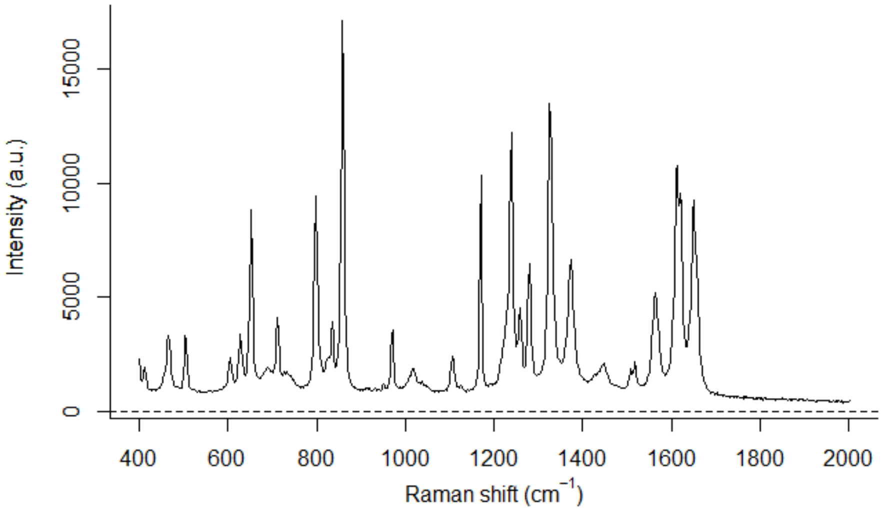

Figure 10 . An example of a paracetamol (REF) spectrum.

5. Exporting/uploading data

5.1. Each original **un-processed single spectrum** should be saved to ASCII file, which should contain two columns of numbers, the first being the Raman shift, the second being the intensity:

```
150.02958817
151.14359742.1
152.25659679.4
153.36959670.6
154.48257664.4
155.59455032.7
156.706…
```

5.2. The **filename** should be structured as follows:

[lab-code]_[substrate]_[laser]_[sample]_[replica(inter)]_[replica(intra)].txt

(examples: P01_sAu_785_C4_1_c.txt; P05_cAg_532_C2_1)

where:

- ***lab-code*** can be **P01, P02, P03, …P20**: the lab-codes will be individually assigned to each Participant by the Round Robin coordinators, to maintain data anonymity;

- ***substrate*** can be **sAu**, **sAg** (solid Au or Ag substrates), **cAu** or **cAg** (colloidal Au or Ag substrates)

- ***laser*** can be **785**, **532** or **514**

- ***sample*** is the sample label, and it can be **C0, C1, C2, …C9** or **X1, X2, …X5**

- ***replica (inter)*** is the inter-substrate replica for the same sample (i.e. number of SOLID substrate or number of batch for the COLLOIDAL substrates) and can be **1, 2** or **3**

(examples of files from different inter-substrate replicas for the same sample: P05_cAg_532_X2_1, P05_cAg_532_X2_2, P05_cAg_532_X2_3)

- ***replica (intra)*** is the intra-substrate replica for the same substrate (only used for SOLID substrates) and can be **a, b** or **c**

(examples of files from different intra-substrate replicas for the same sample: P01_sAu_785_X4_1_a.txt; P01_sAu_785_X4_2_b.txt; P01_sAu_785_X4_3_c.txt)

IMPORTANT: please take care when naming files: correct file naming is crucial, since all meta-data will be derived from the filename, and incorrect filenames will lead to large errors in the results.

5.3. FILES WITH PARACETAMOL (REF) SPECTRA: As indicated in section 4.3 (p. 17) a spectrum of REF (paracetamol) should be collected for each method (i.e. laser/substrate combination) according to the instructions. Then, you should upload it together with the other data, using the following naming scheme:

[lab-code]_[substrate]_[laser]_REF.txt

(examples: P01_sAu_785_REF.txt; P05_cAg_532_REF.txt)

5.4. EXPERIMENTAL DETAILS AND NOTES: For each method please upload an ASCII txt file with the experimental details and notes/remarks. The file should be structures as follows:

```
instrument:
(answer)
collection optics:
(answer)
laser power at the sample:
(answer)
exposure time (s):
(answer)
accumulations (N):
(answer)
additional notes:
(answer)
```

and it should be named as:

[lab-code]_[substrate]_[laser]_methods.txt

(examples: P01_sAu_785_methods.txt; P05_cAg_532_methods.txt)

5.5. Once collected and saved as individual files (formatted as in 5.2 and 5.a) named as in 5.2 and 5.3, data should be **sent to a shared folder via a private Dropbox link** (University of Trieste account, only accessible by the Round Robin Coordinator), which will be communicated to Participants in due time.

Raman4Clinics – Work Group 1

Interlaboratory study

S2 - Data Analysis Protocol (DAP)

Cautionary statement

IMPORTANT: We recommend **inspection** of **data** (e.g. visual inspection of spectra) after each stage of data pre-processing and analysis to spot any adverse effects on the data sets. **All decisions should be taken by *a priori* spectroscopic knowledge of the system**. Although such a strategy may not be the optimal one, it will lead to a better model performance.

Remarks on software to be used

This is a software-independent protocol, i.e. it describes the various operations to be done on data in such a way that they can be carried out using different software and applications. Various data analysis software programs and packages exist, ranging from those for general-purpose use to those targeting specific data analysis tasks. At University of Trieste, the site where the centralized data analysis for the ILS has been made, the analysis was performed using the R statistical computing environment (http://www.r-project.org/) version 3.5.1. Throughout the protocol, metrics, parameters, and approaches that may be somewhat different in other software were explained wherever possible.

Raman4Clinics – Work Group 1

Interlaboratory study

1. DATA IMPORT/LOADING

Timing: depends on the import method and on the software used.

NOTE: Data is available as original files (one file per spectrum) in the RRdata folder, as well as a single CSV file (RRspectra.csv) in which each row is a spectrum. For more information see the document "Data overview".

1.1. Import data of a single dataset (i.e. one method from one lab) into the selected software, making sure all the metadata available in the filenames (i.e. labcode, substrate, laser, method, sample, type, concentration, batch, replica) is attached to each spectrum.

1.2. Carefully inspect the data checking for anomalies, poor-quality spectra (e.g. missing data) and artefacts (e.g. cosmic rays)

2. DATA PRE-PROCESSING

Timing: 5-10 min (depending on the size/kind of the data set. e.g. colloids/solid substrates).

The main goal of this step is to improve the accuracy of the study by minimizing variation within the data that does not pertain to the analytical information.

[SMOOTHING, DOWNSAMPLING AND SELECTION OF SPECTRAL RANGE FOR ANALYSIS]

NOTE: The wavelength axis of the raw spectra is often not evenly spaced (the data points spacing ranges between less than 1 wavenumber to several wavenumbers). Furthermore, it would be good to trade some spectral resolution for higher signal to noise ratio, and remove the uninformative part of the spectra. These three issues were addressed by interpolating and smoothing the wavenumber axis by a Local Polynomial Regression Fitting (LOESS) function to a specific wavenumber region.

2.1. Smooth, down-sample and restrict to the ~400 - ~1650 cm^{-1} wavenumber range by applying a LOESS function[5] to obtain a uniform data step of 3 cm^{-1}.

[5] Interpolation is obtained by locally fitting by weighted least squares. That is, for the fit at point x, the fit is made using points in a neighborhood of x, weighted by their distance from x (with differences in 'parametric' variables being ignored when computing the distance). The size of the neighborhood is controlled by α = 0.75. The neighborhood includes proportion α of the points, and these have tri-cubic weighting (proportional to $\left(1-\left(\frac{dist}{maxdist}\right)^3\right)^3$.

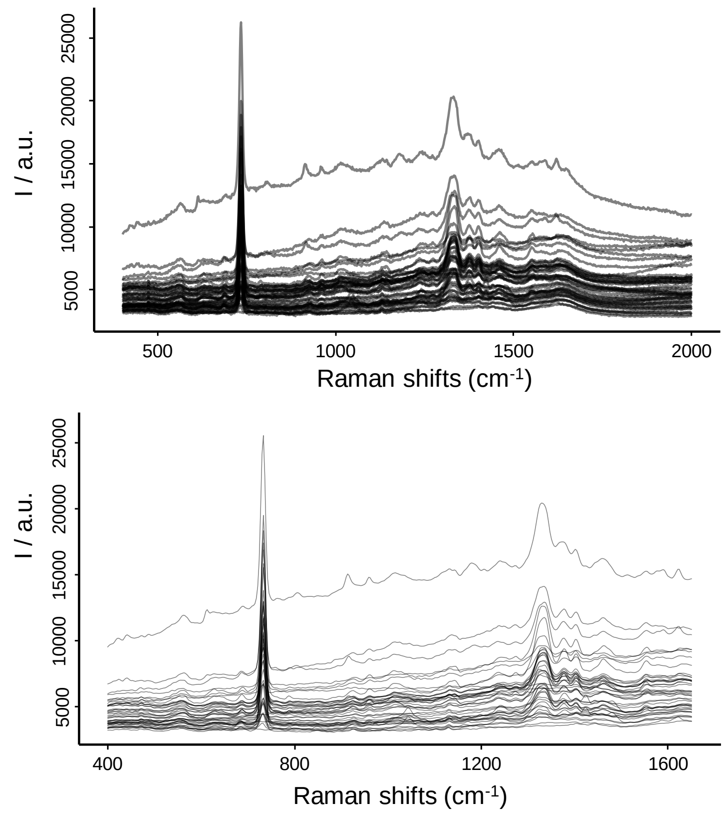

Figure 1 Spectra before and after loess.

[BASELINE CORRECTION AND NORMALIZATION]

NOTE: Extended Multiplicative Signal Correction (EMSC) performs model-based background correction and normalization of spectra, handling variations in scaling, polynomial baselines and interferents. The EMSC methodology and extensions have been described in the EMSC Tutorial of Afseth and Kohler (Afseth and Kohler 2012).

2.2. Correct all the spectra for baseline and intensity normalization applying the Extended Multiplicative Signal Correction (EMSC), by following these steps:

2.2.1. Prepare a spectrum consisting of the average of all pre-processed C0 spectra

2.2.2. Fit and subtract a 2nd order polynomial baseline from the mean C0 spectrum

2.2.3. Use this baseline-subtracted mean C0 spectrum as *customized reference* for the EMSC algorithm. (This is important to ensure that zero signals will be truly zeros in the calibration)

2.2.4. Perform spectral correction on all spectra (C0-C9) using EMSC procedure with polynomials up to the second degree[6]

[Checkpoint] A visual check should be performed to assure that C0 spectra are flat. (Figure 12)

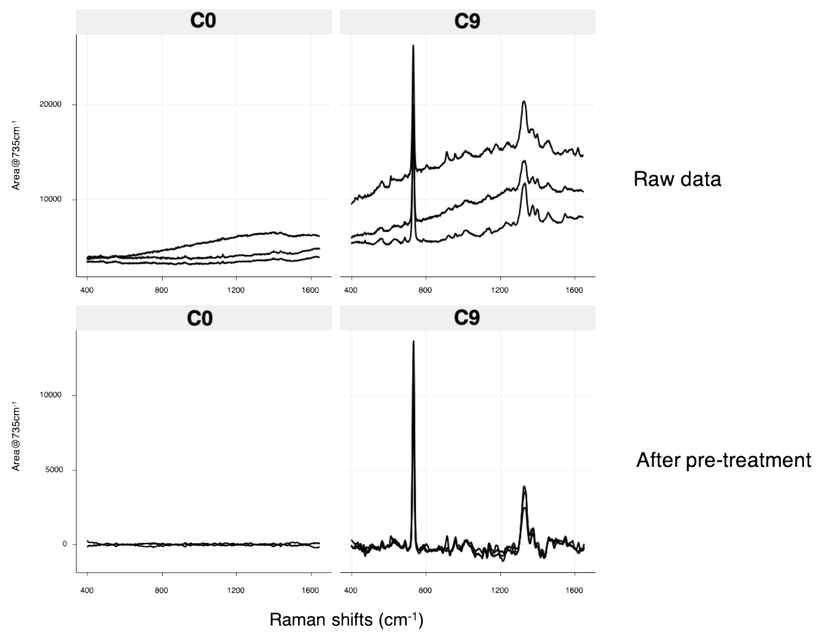

Figure 2 Spectra before and after preprocessing for the Blank (C0) and for the highest adenine concentration (C9).

[6] A R package (EMSC) and a MATLAB graphical user interface for EMSC with several extensions are freely available (https://nofimamodeling.org/). Other implementations of EMSC are also available through Quasar (free, https://quasar.codes) or several commercial software packages.

3. CALIBRATION

Timing: 15-30 min

[AREA CALCULATION]

3.1. Integrate the area of SERS intensity between 715 and 750 cm^{-1} for the specific ring breathing Raman mode of adenine (Figure 13).

3.2. Calculate the average area (A) for each concentration (c).

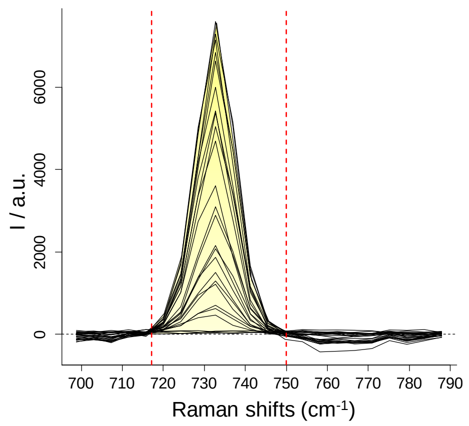

Figure 3 Calculation of the integrated intensity of the adenine

[DATA FRAME CREATION]

3.3. Create two separate data frames (one for the calibration, train set, and one for the validation, test set, of the model), with the average areas (A), and adenine concentrations (C0-9 and X1-5, respectively) (Figure 14).

Table 1 TRAIN set

c	A
0	0
0.2	3412
0.4	5380
0.6	8850
0.8	12436
1	20266
1.2	28740
1.4	27240
1.6	30457
1.8	43381

Table 2 TEST set

X	A
0.23	3522
0.61	8862
1.03	20300
1.4	27246
1.72	43389

Figure 14 Example of TRAIN and TEST tables.

[DATA PLOTTING]

3.4. Plot the average areas against the adenine concentration in the Train set to see if a relationship (a correlation) exists between them (Figure 15). The convention is to plot the instrument response data (A) on the y-axis and the values for the standards (c) on the x-axis.

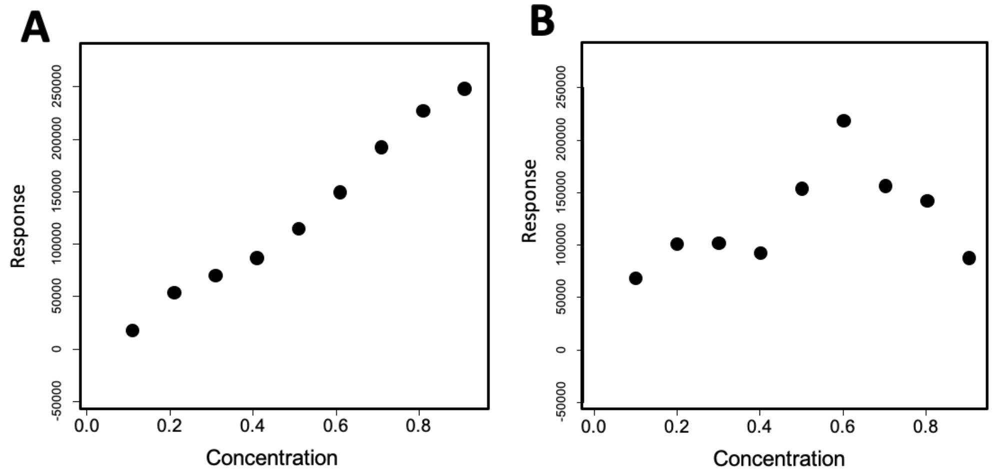

Figure 4 Two examples of concentration vs response plot. The plot of the left (A) is an example of a linear correlation.

[Checkpoint] Inspect the plot for possible extreme values of both c (high leverage points) and A (outliers).

[REGRESSION ANALYSIS]

NOTE: Let us assume that the relationship between A and c is reasonably linear, thus represented namely by a straight-line, there are two obvious ways to fit a linear calibration line for predicting c from A. The so-called "classical" calibration approach would fit a regression line of A on c, while the "inverse" regression would treat c as the response and A as the regressor, fitting a regression line of c on A. Both regression, direct or indirect calibration, are carried out by least squares minimization according to Gauss.

Let (x_i, y_i) be the ith pair of A and c values. The least squares criterion estimates α and β by minimizing the residual sum of squares (sum of squared errors), $SSE = \sum_{i=1}^{N}(x_i - \hat{x}_i)^2$ *where* $\hat{x}_i = \beta_0 + \beta_1 y_i$ *are the points on the estimated regression line and are called the fitted values.*

Even if the "classical" calibration yields the best linear unbiased estimate of the calibration curve A (c), it has been proved that predictions with inverse calibration are more precise than those with the classical calibration (Centner et al., 1998).

3.5. Generate a fitted model in the form $c = \beta_0 + \beta_1 A + \varepsilon$ that finds the values for the parameters that best fit the data in the Train set by least squares. Least-squares linear regression can be carried out using most common statistical software. For manual calculation see, for instance, (Ellison *et al*., 2009).

[Checkpoint] Ensure that the x and y data have been correctly assigned! Regression of *c* on *A* is not the same as the regression of *A* on *c* (except in the highly improbable case where all the points lie exactly on a straight line).

3.6. [OPTIONAL] Calculate the uncertainty associated to the calibration line, and plot the confidence and prediction bands[7] (Figure 16A). It is also useful to obtain a plot of the residuals[8] (Figure 16B).

[7] Uncertainty in a linear regression relationship can be expressed by a 95% confidence interval (green band) and 95% prediction interval (red band). Confidence interval is a range of values that is likely to include the slope and the intercept, with a given confidence level (usually 95%). Prediction interval provides information about a range of values that will contain a single future response value with a given confidence level. It is always wider than confidence interval because it considers the spread in the data and the uncertainty in the model parameters. Unlike confidence intervals, which are accurate when the sampling distribution of the estimator is close to normal, which usually occurs in sufficiently large samples, the prediction interval is accurate only when the errors are close to normal, which is not affected by sample size.

[8] When regression assumptions are met, the plot should have zero mean, constant spread and no global trends: the residuals should be scattered approximately randomly around zero, with no trend in the spread of residuals with concentration.

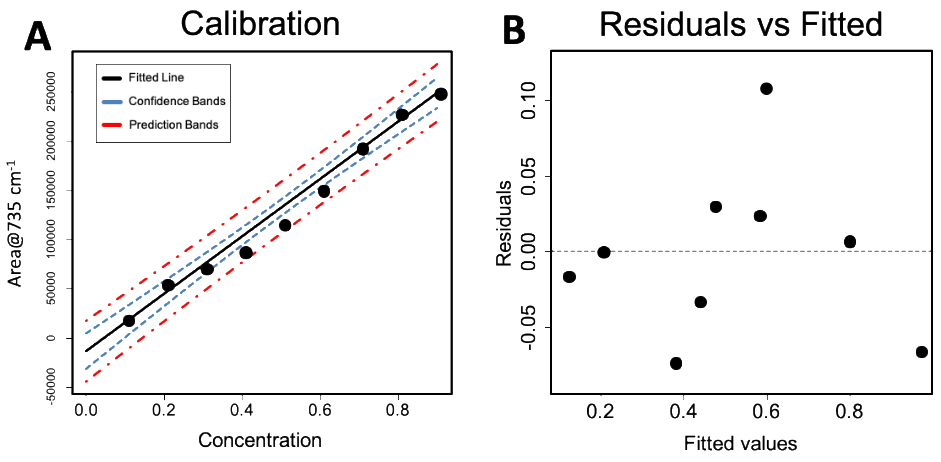

Figure 16. The regression line is shown, together with the confidence (green) and prediction (red) bands, both with a confidence level of 95%. B. Residual plots for the fit.

3.7. [OPTIONAL] Calculating and Interpreting Regression Statistics

The (Pearson product-moment) correlation coefficient **r,** can be obtained from software or calculated using the equation

$$r = \frac{\sum_{i=1}^{n}[(x_i - \bar{x})\,(y_i - \bar{y})]}{\sqrt{[\sum_{i=1}^{n}(x_i - \bar{x})^2][\sum_{i=1}^{n}(y_i - \bar{y})^2]}}$$

The value of r will be in the range ±1. The closer $|r|$ is to 1, the stronger is the correlation between the variables. *r* can be usefully interpreted as indicative of good linearity only when data are reasonably evenly distributed along the x-axis, with no serious anomalies.

[Checkpoint] Correlation coefficients are simple to calculate, but are easily very misinterpreted. The calibration curve must always be plotted and inspected by eye. If not, a straight-line relationship might wrongly be deduced from the calculation of *r*. A *r* value close to 0 does not necessarily mean that there is no relationship; a useful non-linear relationship would not necessarily lead to high linear correlation coefficients. For a simple linear regression, the coefficient of determination r^2 is the square of correlation coefficient *r* between the observed outcomes and the observed predictor values. The r^2 coefficient of determination is a statistical measure of how well the regression predictions approximate the real data points. An r^2 of 1 indicates that the regression predictions perfectly fit the data[9].

[9] The F-test of overall significance is the hypothesis test for this relationship. In a calibration experiment, the R^2 should be significantly different from zero as it is essential that the y and x values are highly correlated. The p-value should therefore be

[OPTIONAL] [ESTIMATION OF LOD VIA A CALIBRATION APPROACH]

NOTE: The limit of detection (LOD) and the limit of quantification (LOQ) are key parameters characterizing the performance of the whole test method. The selection of the procedure for estimation of LOD and LOQ primarily depends on legal requirements, as well as availability of blank samples, the presence of "noise" in the spectra and its applicability for calculations, or the practicability of calibration experiments. The presented statistical-mathematical approach is based on elements taken mainly from DIN 32645:2008-11 (DIN 32645:2008-11 2008) and ISO 11843-2:2000 (ISO TC69/SC6 2010), with the following assumptions/conditions (which, however, in practice are frequently not fully met):

- *normal distribution of the analytical response (signal) of blank and calibration samples;*
- *homoscedasticity (homogeneity of variances) over the calibrated range;*
- *replicate analyses of blank and calibration samples are independent;*
- *linearity between the analytical response and the analyte concentration close to the LOQ;*
- *probabilities for both false positive and false negative decisions of 0.05 are appropriate in the areas covered by the scope of this guidance document*

3.8. Estimate the LOD by using the following equation

$$LOD = 2 * t_{\alpha,\nu} * \frac{s_{y,x}}{b} * \sqrt{\frac{1}{m} + \frac{1}{p*q} 1.1 + \frac{\bar{x}^2}{\sum_{i=1}^{n}(x_i - \bar{x})^2}}$$

where

$s_{y,x}$	is the standard deviation of the residuals
b	is the slope of the calibration curve
x_i	is the value of the analyte concentration at calibration level *i* (e.g. for C1, C2,…C9)
$\bar{x}$	is the mean analyte concentration computed over all calibration levels
$t_{\alpha,\nu}$	is the value from t-distribution for probability level α (one-sided test) and $\nu = (p * q) - 2$ degrees of freedom, with *p* being the number of calibration levels, and *q* the number of replicates at each calibration level
m	is the number of replicate analyses

very small (far less than the usual 0.05) and the F value should be very much greater than the critical value if the calibration is to be useful.

NOTE: For the experimental design specific to this RRT, the equation above is reduced to:

$$LOD = 3.8 * \frac{s_{y,x}}{b} * \sqrt{1.1 + \frac{\bar{x}^2}{\sum_{i=1}^{n}(x_i - \bar{x})^2}}$$

3.7. Estimate the LOQ by using the following equation:

$$LOQ = 3.3 * LOD$$

NOTE: Depending on the definition of LOQ, ratios between LOQ and LOD of about 2, 3.3, or even 1 are possible.

4. MODEL VALIDATION

Timing: 15 min

[PREDICTION OF CONCENTRATION VALUES OF TEST SAMPLES]

NOTE. If, after plotting the data and examining the regression statistics, the calibration data are judged to be satisfactory, the estimated intercept α and the slope β of the calibration line can "directly" be used to estimate the concentration of the analyte in Test set.

4.1. Estimate the unknown X using $\hat{X} = a + bA$

4.2. [OPTIONAL] Calculate the confidence interval for the prediction of the unknown concentrations.

[CALCULATION OF RMSEP AND BIAS]

4.3. Estimate the root mean square error of prediction (RMSEP) and BIAS, defined as follow:

$$RMSEP = \sqrt{\sum_{i=1}^{N} \frac{(\hat{c}_i - c_i)^2}{N}}$$

$$BIAS = \sum_{i=1}^{N} \frac{\hat{c}_i - c_i}{N}$$

Where $\hat{c}_i$ and c_i are the predicted and assigned reference values for the sample *i* and *N* is the number of samples in the TEST set.

[NORMALIZATION OF RMSEP AND BIAS]

4.4. Normalize RMSEP and BIAS with respect to different concentration ranges as follows:

$$NRMSEP = \frac{RMSEP}{c_{max} - c_{min}}$$

$$NBIAS = \frac{BIAS}{c_{max} - c_{min}}$$

Where c_{max} and c_{min} are the maximum and minimum concentration (reference) values for the test samples.

S3 - “Supplementary Figures“

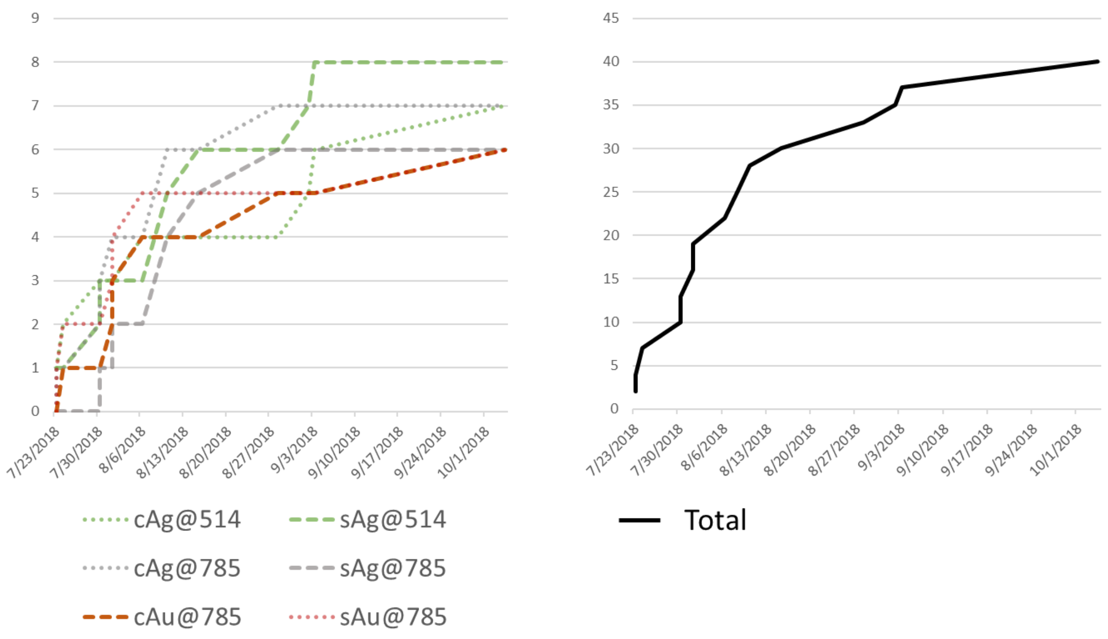

Figure S1. Data Upload

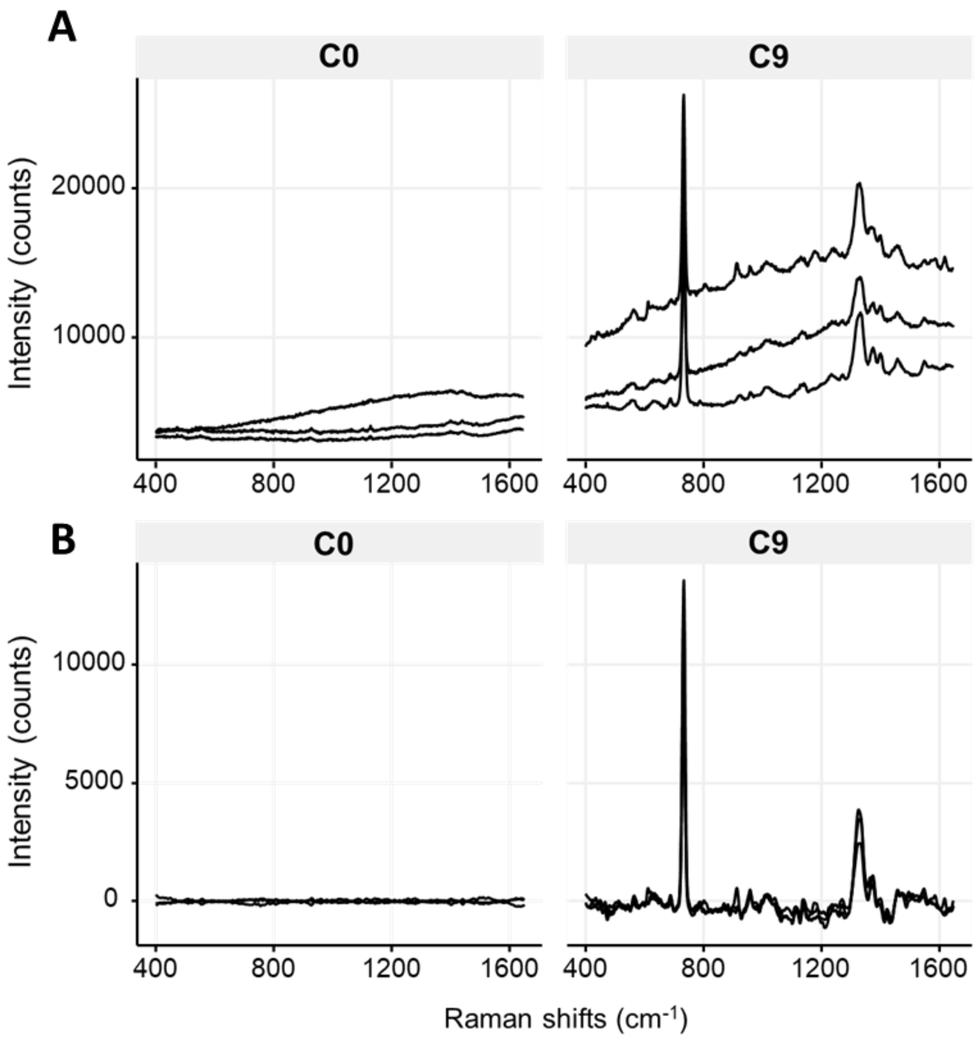

Figure S2. Effect of preprocessing. SERS signal of PBS (C0) and adenine (C9) before (A) and after (B) EMSC.

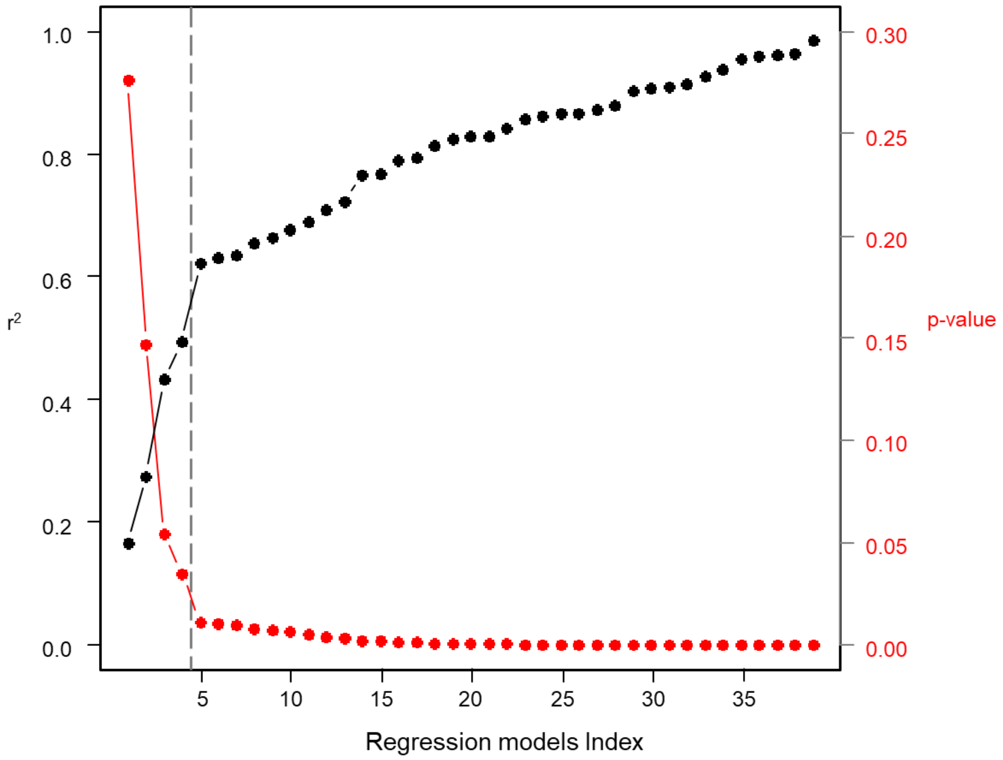

Figure S3. Regression models' evaluation. Each point represents a calibration curve. Models with $r^2 < 0.6$ and p-values >0.01 were not selected for the final report.

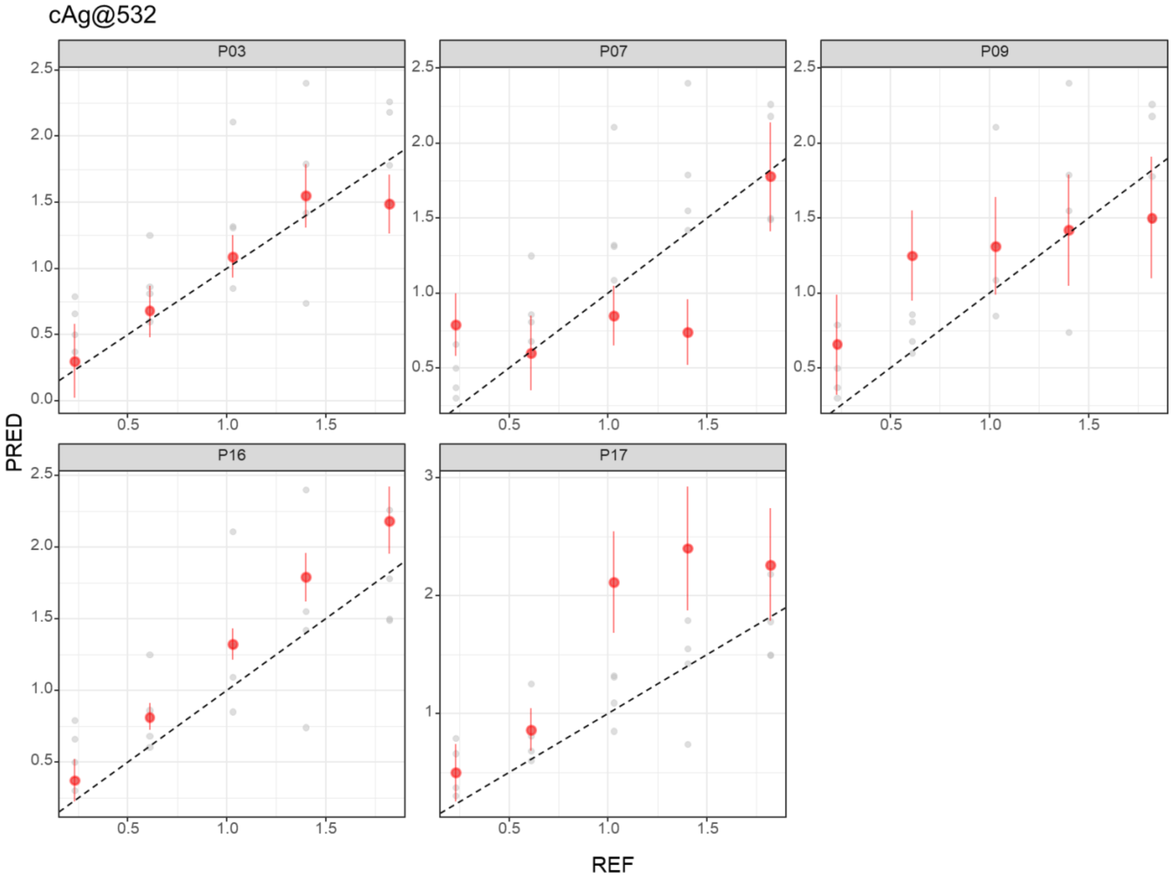

Figure S4. Plot of estimated values against the actual values for the test set samples of the cAg@532 method. Error bars are the 95% prediction interval on estimated concentrations from calibration model of each laboratory; the dotted line is the line of equality.

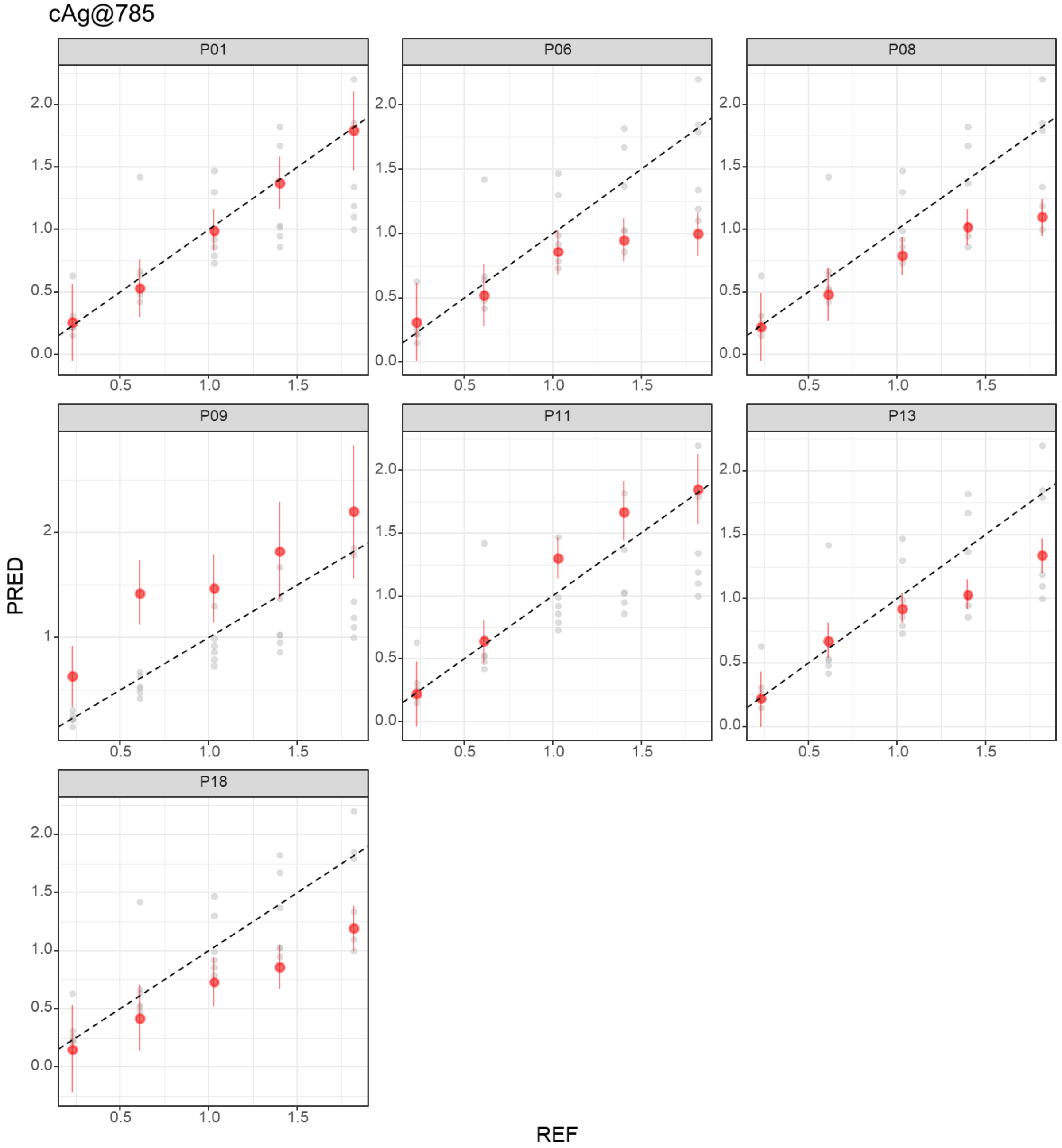

Figure S5. Plot of estimated values against the actual values for the test set samples of the cAg@785 method. Error bars are the 95% prediction interval on estimated concentrations from calibration model of each laboratory; the dotted line is the line of equality.

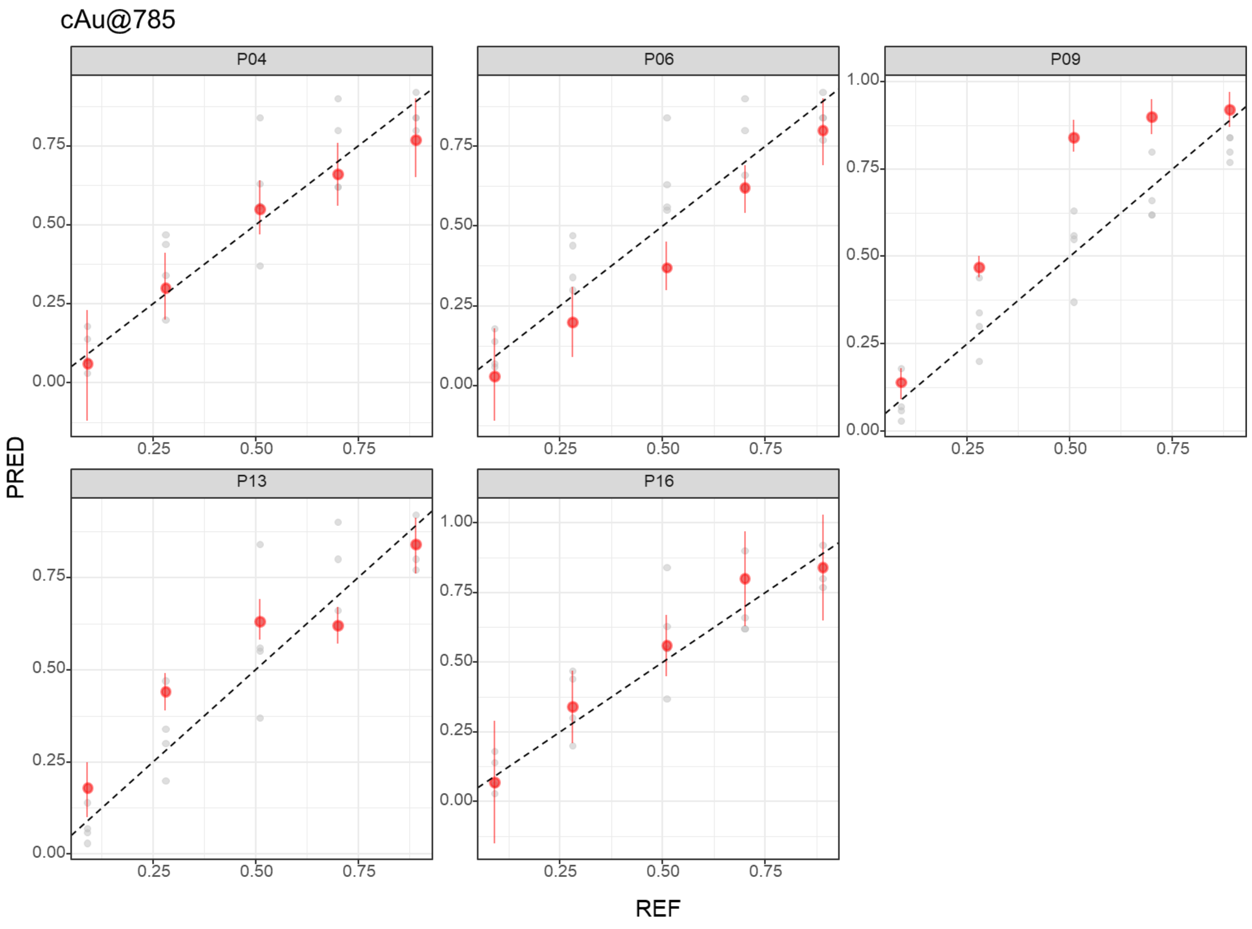

Figure S6. Plot of estimated values against the actual values for the test set samples of the cAu@785 method. Error bars are the 95% prediction interval on estimated concentrations from calibration model of each laboratory; the dotted line is the line of equality.

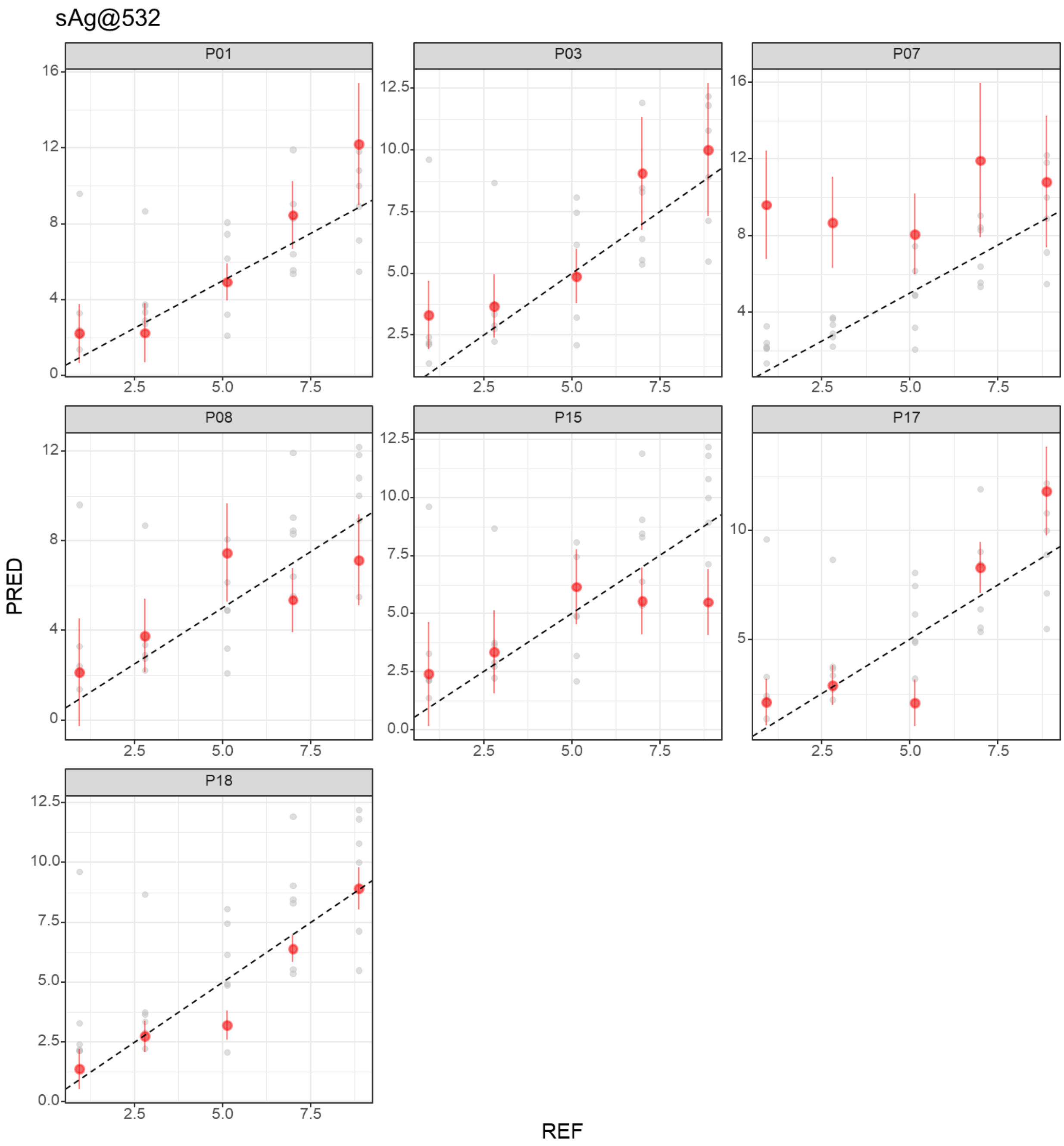

Figure S7. Plot of estimated values against the actual values for the test set samples of the sAg@532 method. Error bars are the 95% prediction interval on estimated concentrations from calibration model of each laboratory; the dotted line is the line of equality.

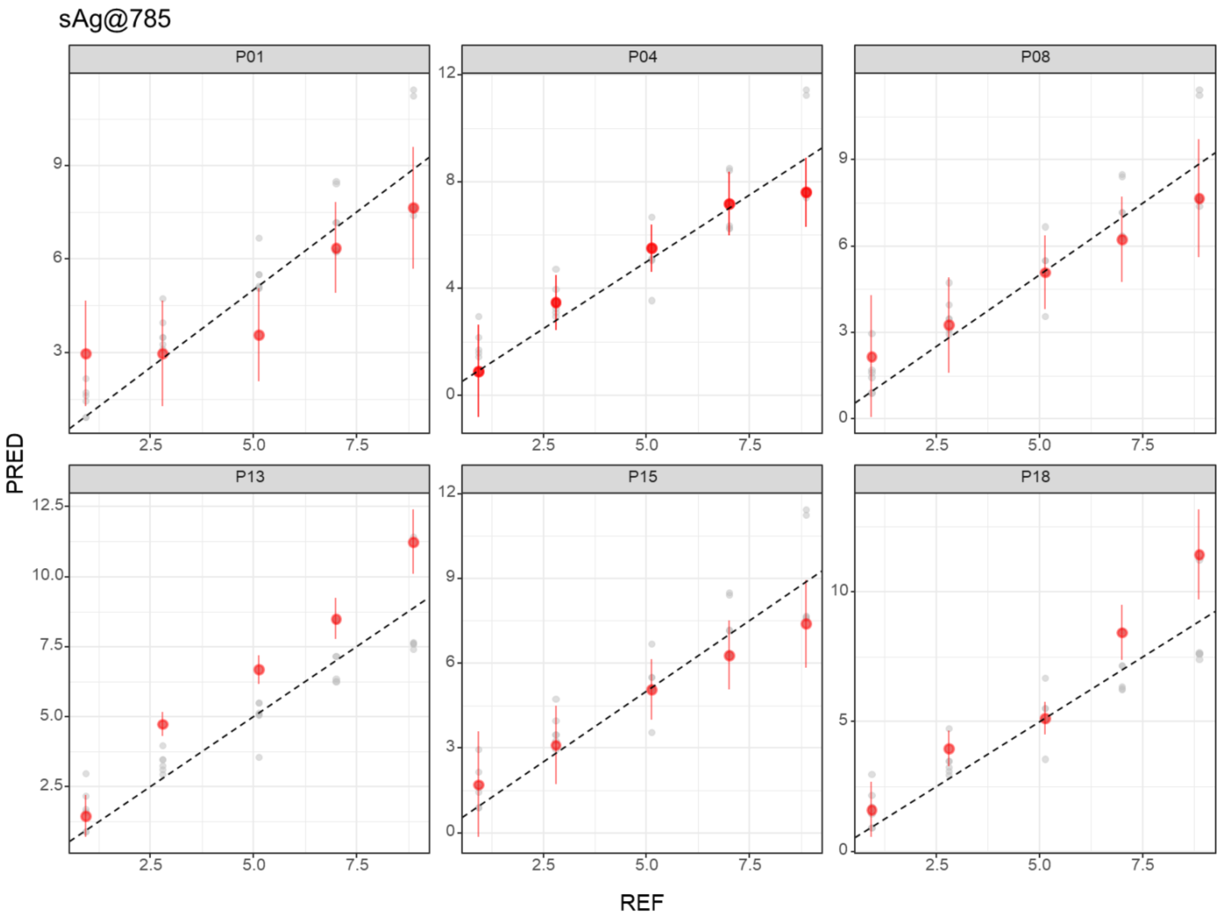

Figure S8. Plot of estimated values against the actual values for the test set samples of the sAg@785 method. Error bars are the 95% prediction interval on estimated concentrations from calibration model of each laboratory; the dotted line is the line of equality.

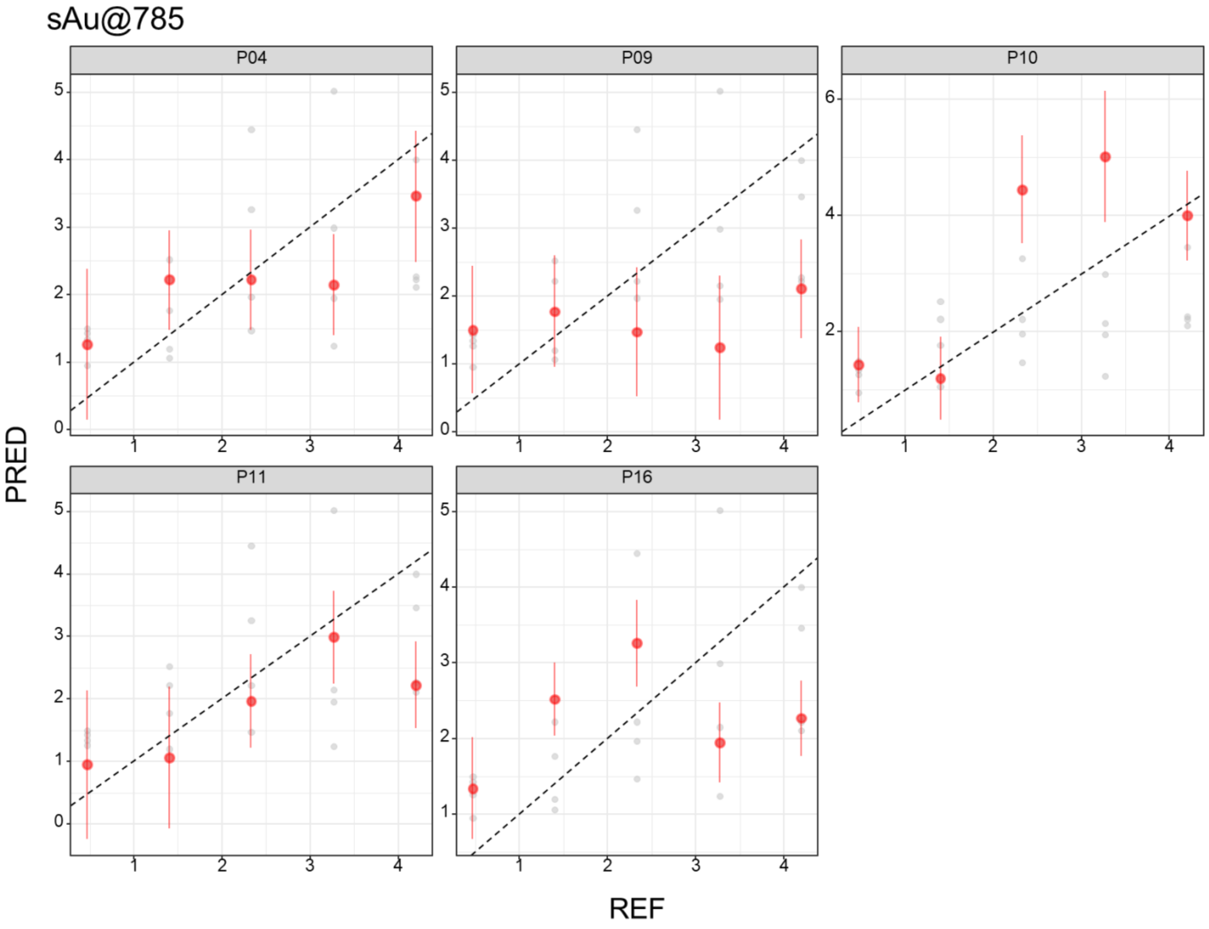

Figure S9. Plot of estimated values against the actual values for the test set samples of the sAu@785 method. Error bars are the 95% prediction interval on estimated concentrations from calibration model of each laboratory; the dotted line is the line of equality.

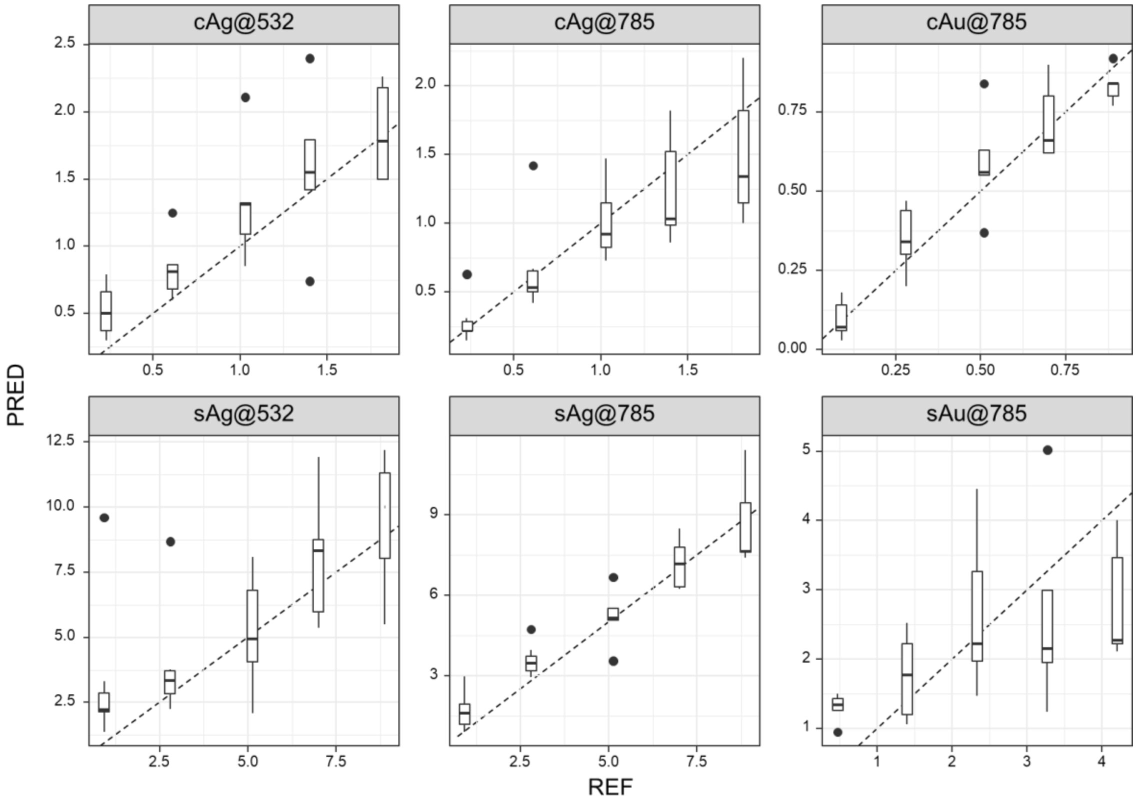

Figure S10. Boxplots of estimated values against the actual values for the test set samples for each method. The line within the box represents the median and the limits of the box are the upper and lower quartiles, so each area spans the interquartile range (IQR in the predictions from different laboratories at the same concentration level; the boxplot whiskers extend up to a maximum of 1.5 times the IQR of the data. The predictions beyond those points (black dots) are considered as outliers.

Raman4Clinics – Work Group 1

Interlaboratory study

S4 - “Supplementary Tables“

Table S1. Adenine concentration levels in the calibration and validation set

ID	*Adenine* *(μM)*			
	cAg@532/ sAg@532	cAg@785/ sAg@785	cAu@785	sAu@785
C0	0	0	0	0
C1	0.2	1	0.1	0.5
C2	0.4	2	0.2	1
C3	0.6	3	0.3	1.5
C4	0.8	4	0.4	2
C5	1	5	0.5	2.5
C6	1.2	6	0.6	3
C7	1.4	7	0.7	3.5
C8	1.6	8	0.8	4
C9	1.8	9	0.9	4.5
X1	0.23	0.93	0.09	0.47
X2	0.61	2.80	0.28	1.40
X3	1.03	5.13	0.51	2.33
X4	1.40	7	0.70	3.27
X5	1.82	8.87	0.89	4.20

Table S2. Prediction results on the validation set for the cAg@532 method.

METHOD	*Laboratory*	*Reference value*	*Predicted value*	*95%CI*	
cAg@532	P03	0.23	0.30	0.02	0.58
	P03	0.61	0.68	0.48	0.87
	P03	1.03	1.09	0.93	1.25
	P03	1.40	1.55	1.31	1.79
	P03	1.82	1.49	1.26	1.71
	P07	0.23	0.79	0.58	1.00
	P07	0.61	0.60	0.35	0.85
	P07	1.03	0.85	0.65	1.05
	P07	1.40	0.74	0.52	0.96
	P07	1.82	1.78	1.41	2.14
	P09	0.23	0.66	0.32	0.99
	P09	0.61	1.25	0.95	1.55
	P09	1.03	1.31	0.99	1.64
	P09	1.40	1.42	1.05	1.79
	P09	1.82	1.50	1.10	1.91
	P16	0.23	0.37	0.22	0.52
	P16	0.61	0.81	0.72	0.91
	P16	1.03	1.32	1.21	1.43
	P16	1.40	1.79	1.62	1.96
	P16	1.82	2.18	1.95	2.42
	P17	0.23	0.50	0.25	0.74
	P17	0.61	0.86	0.68	1.04
	P17	1.03	2.11	1.68	2.54
	P17	1.40	2.40	1.87	2.92
	P17	1.82	2.26	1.78	2.74

Table S3. Prediction results on the validation set for the cAg@785 method.

METHOD	*Laboratory*	*Reference value*	*Predicted value*	*95%$_{CI}$*	
cAg@785	P01	0.23	0.26	-0.05	0.56
	P01	0.61	0.53	0.3	0.76
	P01	1.03	0.99	0.83	1.16
	P01	1.40	1.37	1.16	1.58
	P01	1.82	1.79	1.47	2.1
	P06	0.23	0.31	0.01	0.61
	P06	0.61	0.52	0.28	0.76
	P06	1.03	0.86	0.68	1.03
	P06	1.40	0.95	0.78	1.12
	P06	1.82	1.00	0.83	1.16
	P08	0.23	0.22	-0.05	0.49
	P08	0.61	0.48	0.27	0.69
	P08	1.03	0.79	0.63	0.94
	P08	1.40	1.02	0.87	1.16
	P08	1.82	1.10	0.95	1.24
	P09	0.23	0.63	0.34	0.92
	P09	0.61	1.42	1.12	1.73
	P09	1.03	1.47	1.14	1.79
	P09	1.40	1.82	1.36	2.29
	P09	1.82	2.20	1.56	2.83
	P11	0.23	0.22	-0.04	0.48
	P11	0.61	0.64	0.46	0.81
	P11	1.03	1.30	1.14	1.47
	P11	1.40	1.67	1.44	1.91
	P11	1.82	1.85	1.57	2.13
	P13	0.23	0.22	0.00	0.43
	P13	0.61	0.67	0.54	0.81
	P13	1.03	0.92	0.81	1.04
	P13	1.40	1.03	0.92	1.15
	P13	1.82	1.34	1.20	1.47
	P18	0.23	0.15	-0.22	0.53
	P18	0.61	0.42	0.14	0.71
	P18	1.03	0.73	0.52	0.94
	P18	1.40	0.86	0.67	1.05
	P18	1.82	1.19	1.00	1.39

Table S4. Prediction results on the validation set for the cAu@785 method.

METHOD	Laboratory	Reference value	Predicted value	$95\%_{CI}$	
cAu@785	P04	0.09	0.06	-0.12	0.23
	P04	0.28	0.30	0.20	0.41
	P04	0.51	0.55	0.47	0.64
	P04	0.70	0.66	0.56	0.76
	P04	0.89	0.77	0.65	0.90
	P06	0.09	0.03	-0.11	0.18
	P06	0.28	0.20	0.09	0.31
	P06	0.51	0.37	0.30	0.45
	P06	0.70	0.62	0.54	0.69
	P06	0.89	0.80	0.69	0.90
	P09	0.09	0.14	0.09	0.18
	P09	0.28	0.47	0.44	0.50
	P09	0.51	0.84	0.80	0.89
	P09	0.70	0.90	0.85	0.95
	P09	0.89	0.92	0.87	0.97
	P13	0.09	0.18	0.10	0.25
	P13	0.28	0.44	0.39	0.49
	P13	0.51	0.63	0.58	0.69
	P13	0.70	0.62	0.57	0.67
	P13	0.89	0.84	0.76	0.91
	P16	0.09	0.07	-0.15	0.29
	P16	0.28	0.34	0.21	0.47
	P16	0.51	0.56	0.45	0.67
	P16	0.70	0.80	0.63	0.97
	P16	0.89	0.84	0.65	1.03

Table S5. Prediction results on the validation set for the sAg@532 method.

METHOD	Laboratory	Reference value	Predicted value	$95\%_{CI}$	
sAg@532	P01	0.93	2.21	0.66	3.76
	P01	2.80	2.24	0.70	3.78
	P01	5.13	4.93	3.94	5.92
	P01	7.00	8.46	6.68	10.23
	P01	8.87	12.2	8.97	15.42
	P03	0.93	3.30	1.91	4.69
	P03	2.80	3.67	2.38	4.96
	P03	5.13	4.88	3.77	5.99
	P03	7.00	9.05	6.77	11.33
	P03	8.87	10.01	7.31	12.71
	P07	0.93	9.61	6.78	12.45
	P07	2.80	8.68	6.31	11.06
	P07	5.13	8.08	5.99	10.18
	P07	7.00	11.92	7.90	15.95
	P07	8.87	10.81	7.37	14.25
	P08	0.93	2.14	-0.27	4.54
	P08	2.80	3.76	2.12	5.40
	P08	5.13	7.46	5.27	9.66
	P08	7.00	5.36	3.93	6.78
	P08	8.87	7.14	5.12	9.17
	P15	0.93	2.41	0.16	4.65
	P15	2.80	3.35	1.57	5.14
	P15	5.13	6.16	4.56	7.76
	P15	7.00	5.55	4.11	7.00
	P15	8.87	5.50	4.07	6.94
	P17	0.93	2.13	1.06	3.20
	P17	2.80	2.90	1.99	3.82
	P17	5.13	2.09	1.02	3.17
	P17	7.00	8.31	7.15	9.48
	P17	8.87	11.82	9.77	13.87
	P18	0.93	1.37	0.52	2.21
	P18	2.80	2.74	2.09	3.38
	P18	5.13	3.21	2.61	3.80
	P18	7.00	6.40	5.85	6.96
	P18	8.87	8.92	8.03	9.81

Table S6. Prediction results on the validation set for the sAg@785 method.

METHOD	*Laboratory*	*Reference value*	*Predicted value*	*95%$_{CI}$*	
sAg@785	P01	0.93	2.97	1.27	4.67
	P01	2.80	2.96	1.26	4.66
	P01	5.13	3.56	2.06	5.05
	P01	7.00	6.36	4.90	7.83
	P01	8.87	7.64	5.69	9.59
	P04	0.93	0.91	-0.82	2.64
	P04	2.80	3.48	2.45	4.51
	P04	5.13	5.51	4.61	6.40
	P04	7.00	7.17	5.99	8.35
	P04	8.87	7.60	6.31	8.89
	P08	0.93	0.91	-0.82	2.64
	P08	2.80	3.48	2.45	4.51
	P08	5.13	5.51	4.61	6.40
	P08	7.00	7.17	5.99	8.35
	P08	8.87	7.60	6.31	8.89
	P13	0.93	2.17	0.05	4.30
	P13	2.80	3.26	1.61	4.91
	P13	5.13	5.09	3.81	6.37
	P13	7.00	6.24	4.76	7.73
	P13	8.87	7.67	5.62	9.71
	P15	0.93	1.45	0.70	2.20
	P15	2.80	4.74	4.30	5.17
	P15	5.13	6.68	6.16	7.20
	P15	7.00	8.50	7.76	9.24
	P15	8.87	11.24	10.09	12.39
	P18	0.93	1.71	-0.15	3.58
	P18	2.80	3.11	1.73	4.49
	P18	5.13	5.07	4.00	6.14
	P18	7.00	6.28	5.06	7.50
	P18	8.87	7.40	5.85	8.94

Table S7. Prediction results on the validation set for the sAu@785 method.

METHOD	*Laboratory*	*Reference value*	*Predicted value*	*95%$_{CI}$*	
sAu@785	P04	0.47	1.26	0.15	2.38
	P04	1.40	2.22	1.48	2.95
	P04	2.33	2.22	1.48	2.96
	P04	3.27	2.15	1.40	2.9
	P04	4.20	3.46	2.48	4.43
	P09	0.47	1.50	0.56	2.44
	P09	1.40	1.77	0.95	2.6
	P09	2.33	1.47	0.52	2.42
	P09	3.27	1.24	0.18	2.3
	P09	4.20	2.11	1.38	2.83
	P10	0.47	1.43	0.79	2.08
	P10	1.40	1.20	0.49	1.91
	P10	2.33	4.45	3.53	5.38
	P10	3.27	5.02	3.89	6.15
	P10	4.20	4.00	3.22	4.77
	P11	0.47	0.95	-0.24	2.14
	P11	1.40	1.06	-0.07	2.2
	P11	2.33	1.97	1.22	2.72
	P11	3.27	2.99	2.25	3.73
	P11	4.20	2.22	1.53	2.92
	P16	0.47	1.34	0.67	2.02
	P16	1.40	2.52	2.04	3.00
	P16	2.33	3.26	2.68	3.83
	P16	3.27	1.95	1.42	2.48
	P16	4.20	2.27	1.77	2.76

Table S8. Figures of merit for different SERS methods.

Method	*Calibration Range (µM)*	*RMSEP (µM)*	*SEP (µM)*	*BIAS (µM)*
cAg@532	0.23 – 1.82	0.43	0.39	0.21
cAg@785	0.23 – 1.82	0.35	0.35	-0.08
cAu@785	0.09 – 0.89	0.11	0.11	0.02
sAg@532	0.93 – 8.87	2.56	2.38	1.02
sAg@785	0.93 – 8.87	1.13	1.11	0.31
sAu@785	0.47 – 4.00	1.18	1.20	-0.09

REFERENCES

Afseth, N. K. and A. Kohler (2012). "Extended multiplicative signal correction in vibrational spectroscopy, a tutorial." *Chemometrics and Intelligent Laboratory Systems* **117**: 92-99.
Centner, V., D. L. Massart and S. de Jong (1998). "Inverse calibration predicts better than classical calibration." *Fresenius' Journal of Analytical Chemistry* **361**(1): 2-9.
DIN 32645:2008-11 (2008). *Chemical analysis - Decision limit, detection limit and determination limit under repeatability conditions - Terms, methods, evaluation (Technical standard)*. Berlin, Deutsches Institut für Normung.
Ellison, S. L. R., V. J. Barwick and T. J. D. Farrant (2009). Regression. *Practical Statistics for the Analytical Scientist: A Bench Guide*, LGC Limited**:** 92-113.
ISO TC69/SC6 (2010). Measurement methods and results: ISO 11843-2:2000 - Capability of detection. *Part 2: Methodology in the linear calibration case.*
